# The Motivation for Cognitive Activities Questionnaire: scale development and validation in French and English

**DOI:** 10.3389/fpsyg.2026.1907732

**Published:** 2026-10-30

**Authors:** Marine Beaudoin, Morgane Schlemmer, Fanny Vallet, Jérôme Blondé, Nathalie Mella, Olivier Desrichard

**Affiliations:** 1 Laboratoire Inter-Universitaire de Psychologie, University Savoie Mont Blanc, Chambéry, France; 2 Faculté de Psychologie et des Sciences de l’Education, University of Geneva, Geneva, Switzerland

**Keywords:** cognitive engagement, expectancy value, motivation, scale validation and development, self-determination

## Abstract

The current research aimed to develop and validate a novel measurement scale, the Motivation for Cognitive Activities Questionnaire (MCAQ), specifically designed to assess individuals’ motivation to engage in everyday cognitive activities. The MCAQ’s development was informed by expectancy-value theory and self-determination theory. Four studies were conducted to validate the MCAQ in both English and French languages. Through exploratory and confirmatory factor analyses, a 32-item scale emerged, encompassing four subscales: perceived capacity, intrinsic motivation, identified motivation, and external motivation, as well as four additional general items assessing motivation for engaging in cognitive activity, taken broadly. The MCAQ demonstrated robust internal consistency and convergent validity, and satisfactory test–retest reliability. Moreover, the scale exhibited satisfactory predictive validity in relation to cognitive performance. The theoretical and practical utility of the MCAQ is discussed.

## 1 Introduction

A comprehensive understanding of cognition requires a multidimensional approach that incorporates motivational factors (Bandura, 1993; Hess, 2022). Indeed, the motivation to engage in cognitive activities is a crucial determinant of cognitive performance (see Forstmeier et al., 2022). A growing body of research highlights how individuals’ motivation for everyday cognitive activities shapes the development and maintenance of functional abilities across the lifespan, particularly in later life (e.g., Hess and Neupert, 2012; Penningroth and Scott, 2007; Queen and Hess, 2018; Vallet et al., 2020). For instance, Forstmeier and Maercker (2008) demonstrated that motivational abilities among older adults are strongly predictive of cognitive aging and may serve as protective factors against cognitive decline.

Despite the growing recognition of the role of motivation in cognitive functioning, existing measurement tools provide only a partial assessment of individuals’ motivation to engage in everyday cognitive activities. The present research addresses this gap by developing and validating the Motivation for Cognitive Activities Questionnaire (MCAQ), a four-factor measurement scale grounded in major contemporary theories of motivation. Here, we report a multi-study validation program designed to establish the instrument’s internal structure and reliability, as well as its association with theoretically related constructs.

### 1.1 Limitations of existing scales of motivation for cognitive activities

The importance of motivation in cognitive processes has led to the development of various measurement scales over the past few decades. Nevertheless, most scales have been designed to assess facets of motivation within specific populations and/or in relation to specific cognitive activities. For example, the Personality in Intellectual Aging Context Inventory evaluates control beliefs related motivation to engage in cognitive tasks among older adults (Lachman et al., 1982). The Motivated Strategies for Learning Questionnaire (Pintrich et al., 1993) and the Academic Motivation Scale (Vallerand et al., 1992) both measure motivation to exert cognitive effort during learning tasks, while the Metamemory in Adulthood questionnaire (Dixon et al., 1988) assesses how individuals evaluate their memory abilities and motivation to achieve good memory outcomes. Similarly, the Creative Trait Motivation scale (Taylor and Kaufman, 2021) captures individual differences in motivation to engage in creative activities across varied domains such as art, science, and everyday life.

While these instruments are valuable within their respective contexts, they are however limited in addressing the variety of cognitive tasks that adults of varying ages encounter in daily life. Moreover, existing scales often focus on specific dimensions of motivation and fail to capture its multidimensional nature. For example, the Need for Cognition (NFC) scale evaluates a dispositional inclination to engage in and derive pleasure from cognitive activities (Cacioppo and Petty, 1982; Cacioppo et al., 1996). As such, it addresses only one aspect of reasons for engaging in cognitive activities, namely intrinsic motivation (i.e., engagement in activities because they are inherently enjoyable and of high personal interest). However, self-determination theory (Deci and Ryan, 2008; Ryan and Deci, 2000, 2020) posits that motivation encompasses additional dimensions, such as identified motivation (i.e., engagement in a task guided by internalized values perceived as personally beneficial) and external motivation (i.e., engagement in a task that is driven by the pursuit of external rewards or the avoidance of punishment), which are not covered by any current measures of motivation for cognitive activities.

Therefore, existing scales contain evident limitations, notably because they are restricted to specific populations (e.g., older adults, students), focus on particular cognitive domains (e.g., creativity, learning), and selectively evaluate limited facets of motivation for cognitive activities (e.g., intrinsic motivation). Consequently, there is a need for a robust measuring instrument that comprehensively assesses individuals’ motivation for various cognitive activities by considering motivation across multiple dimensions. The development of such a tool is pivotal for advancing both research and practical applications. For instance, enhancing motivation to initiate diverse cognitive tasks is beneficial in maintaining functional abilities among older adults (Bielak and Gow, 2023; Queen and Hess, 2018; Stine-Morrow et al., 2022). Thus, an ecologically valid measure that captures motivation for cognitive activities reflective of everyday functioning would be of primary practical interest. The present research is dedicated to the development and validation of a measurement scale specifically designed to achieve this objective.

### 1.2 Theoretical foundations of the MCAQ

The MCAQ, developed and validated in the current research, addressed the limitations of existing measures of motivation toward cognitive activity. It is designed to assess individuals’ motivation to engage in a variety of cognitive activities encountered in everyday life. This represents an individual-differences variable, reflecting a stable disposition to seek out and persist in cognitively demanding tasks, with observable variation across individuals.

The MCAQ integrates well-established dimensions from major theoretical frameworks, most notably self-determination theory and expectancy-value theory. By grounding the scale in these theories, the MCAQ provides a conceptually coherent instrument for assessing motivation toward cognitive activity. Specifically, it is structured around four dimensions of motivation: perceived capacity, intrinsic motivation, identified motivation, and external motivation. The first dimension, namely perceived capacity, evaluates individuals’ beliefs about their ability to perform specific cognitive tasks. This dimension is informed by expectancy-value theory (Eccles and Wigfield, 2020, 2024), which posits that motivation to perform a task comprises two central constructs: subjective value (i.e., the reason why an individual engages in an action), which will be covered by the subsequent dimensions, and expectations (i.e., the perceived personal capacity to engage in an action), which have been extensively shown to be key determinants of task performance (e.g., Beaudoin and Desrichard, 2011, 2017; Eccles and Wigfield, 2023; Jopp and Hertzog, 2007; Valentijn et al., 2006).

Along with perceived capacity, the MCAQ incorporates three additional dimensions that evaluate individuals’ reasons for engaging in an activity, which are derived from self-determination theory (Ryan and Deci, 2000, 2020). These dimensions, which reflect distinct levels of motivation along a continuum of self-determination, include intrinsic motivation, identified motivation, and external motivation. These dimensions significantly influence how one engages in a task. Specifically, self-determination theory argues that intrinsic and identified motivations positively predict cognitive performance and engagement in cognitively demanding tasks, whereas external motivation is negatively associated with these outcomes (Chemolli and Gagné, 2014; Ryan and Deci, 2000; Ryan et al., 2022). Indeed, performance in a task can be improved to the extent individuals find intrinsic satisfaction or personal interest in performing it. Conversely, if an action is undertaken due to external opportunities or constraints, poor performance is more likely to occur. Therefore, the MCAQ assesses individuals’ perceived capacity to perform various cognitive activities, along with their intrinsic, identified, and external motivations for engaging in these activities.

The MCAQ was developed to address several limitations of existing measures of motivation for cognitive activity. First, as outlined above, it assesses multiple motivational dimensions grounded in core theoretical frameworks of motivation. Second, rather than focusing exclusively on a single activity (e.g., learning or memory), it covers a broad range of everyday cognitive activities that individuals commonly perform in daily life. Third, it is designed for use with the general population and is not restricted to any specific demographic or participant group. In this respect, the MCAQ was designed to address important needs in both research and practice by providing a comprehensive measure that can be applied across diverse populations and a wide range of contexts involving cognitively demanding activities.

### 1.3 Overview of the present research

The present research sought to develop and validate the MCAQ through a comprehensive, multi-phase approach. As an initial step, we carried out preliminary scale-development work through which we identified a set of everyday cognitive activities and generated items corresponding to each targeted motivational dimension. Following scale development, we conducted four studies to rigorously validate the MCAQ. Study 1 explored the factor structure of the MCAQ through exploratory factor analyses. To provide stronger evidence for the MCAQ’s dimensionality, Study 2 further examined its factor structure by employing a confirmatory factor analysis. In Study 3, the scale was translated into English and the MCAQ factor structure was examined in both French and English languages. Convergent validity was also examined by correlating the MCAQ with theoretically related scales, and test–retest reliability was established. Finally, Study 4 was designed to examine the MCAQ’s psychometric properties, including its predictive validity in relation to cognitive performance, using data from a large-scale longitudinal survey conducted within the educational domain.

## 2 Scale development

Prior to validating the scale, we conducted an initial scale-development phase. First, we sought to generate an initial pool of everyday cognitive activities that could serve as the basis for item development. Our goal was not to produce a representative inventory of cognitive activities, but rather to identify a diverse set of activities experienced in everyday contexts that could inform the development of the questionnaire. To achieve this, we adopted a data-driven and ecologically grounded approach. This approach was chosen because our aim was to identify cognitive activities as they are experienced in everyday life, rather than relying exclusively on theoretically predefined categories or activities traditionally examined in cognitive research. By starting from participants’ own experiences, we aimed to identify activities that would be familiar and meaningful to individuals from different age groups. In addition, this approach allowed us to identify formulations and examples that would be familiar and easily understood by future respondents from the general population. We recruited a small convenient sample of 11 French-speaking participants from diverse age groups: four young adults (20–30 years), three middle-aged adults (40–60 years), and four older adults (65 years and above). Participants were asked to keep a diary over three consecutive days, in which they documented any experiences involving cognitive activity. Subsequently, three age-specific focus groups were conducted. During these sessions, participants discussed both the activities reported in their diaries and any additional relevant activities that emerged from the group discussions. The purpose of these focus groups was to clarify the nature of the activities reported by participants, to collaboratively generate and refine broader categories of activities based on all activities identified, and to determine illustrative examples that would help potential respondents understand these categories quickly. Based on the diary entries and focus group discussions, we established a list of 12 types of everyday cognitive activities, initially formulated in French. These activities were: (1) “*Analyze and solve problems*”, (2) “*Read short texts*”, (3) “*Perform mental calculations*”, (4) “*Use your sense of direction*”, (5) “*Memorize things*”, (6) “*Recall information*”, (7) “*Plan or organize tasks*”, (8) “*Succeed on mental ability tests*”, (9) “*Engage in creative tasks*”, (10) “*Play mind game*s”, (11) “*Learn new information*”, and (12) “*Write*.” In addition, we developed a generic item reflecting engagement in cognitive activity in a broad sense. Based on the focus group discussions, the expression “Use your brain” emerged as a candidate formulation.

Second, we generated four items for each activity to assess perceived capacity, intrinsic motivation, identified motivation, and external motivation. Following theoretical conceptualizations of these dimensions, perceived capacity items were introduced with “Rate your ability to…”, intrinsic motivation items with “I like/I enjoy…”, identified motivation items with “It is useful to me/I do it for my own good…”, and external motivation items with “I am forced to/I think it is expected of me…”. These items were pilot-tested with a sample of 23 French-speaking undergraduate students. Participants rated each item along two dimensions: “understandability” (1 = completely understandable to 7 = not understandable) and “ease of response” (1 = very easy to answer to 7 = very difficult to answer), with lower scores indicating more favorable evaluations. They also responded to an open-ended question allowing them to provide qualitative feedback. Overall, participants found the items highly understandable (*M* = 1.23, *SD* = 0.28) and relatively easy to answer (*M* = 1.61, *SD* = 0.44). However, the items assessing general cognitive activity (i.e., perceived ability to “use one’s brain”) were perceived as more difficult and less understandable than the others (*M* = 3.13, *SD* = 1.87). Based on this feedback, we refined the wording of items related to general cognitive activity. In particular, the phrasing “*use one’s brain*” was revised to “use one’s brain to engage in activities that require concentration or reflection” to enhance clarity.

Based on this initial development work, we created a preliminary scale consisting of four items (corresponding to the four motivational dimensions) for each of the twelve selected everyday cognitive activities, plus four additional items reflecting general engagement in cognitive activity. In total, this produced 52 items. The subsequent studies were designed to validate this newly developed scale.

The data for Studies 1, 2, and 3 are openly available on the Open Science Framework (https://osf.io/terzf/?view_only=d600ecf1b345465283439c20e2bd6c15). The ProFAN data (Study 4) are available upon request from the authors. The studies were not pre-registered. All measures used in the studies are described in the article. The procedure and objectives of Study 1, 2, and 3 were approved by the Institutional Review Board (IRB) at the authors’ institution (N°: PSE.20170803.18). Prior to data collection, we aimed to recruit a minimum of 500 participants for each study, a sample size considered optimal for scale validation (Comrey and Lee, 1992; Guadagnoli and Velicer, 1988). A full description of the MCAQ and its administration protocol (in both English and French) can be found in the Supplementary material.

## 3 Study 1

The objective of Study 1 was to examine the factorial structure of the French version of the MCAQ. Building on our preliminary scale-development work, which identified 12 everyday cognitive activities and generated items for the four theoretically grounded motivational dimensions, this study aimed to empirically evaluate whether the items group together in accordance with the theoretical structure. Specifically, we conducted exploratory factor analyses (EFA) on all items corresponding to specific cognitive activities. The results of this study provide a foundational understanding of the scale’s structure and inform the selection and refinement of items for subsequent validation steps.

### 3.1 Methods

#### 3.1.1 Participants and procedure

A total of 502 French-speaking participants were recruited through the research panel platform FouleFactory. Participants’ ages ranged from 18 to 75 years (*M* = 37.50, *SD* = 11.53), including 261 women (52.0%) and 241 men (48.0%). Most participants had completed at least 2 years of higher education (64.0%), while 26.7% had completed secondary education and 9.4% had a vocational or lower-level qualification. Regarding household income, 41.4% reported an annual pre-tax household income below €20,000, 40.2% between €20,000 and €40,000, and 18.3% above €40,000. The study was conducted online via the Qualtrics platform. After providing informed consent, participants completed the MCAQ items in French. Upon completion, they were thanked for their participation and compensated financially for their time.

#### 3.1.2 Measures

Participants completed the MCAQ, which included the full set of items and cognitive activities generated during the scale-development phase. Items assessing motivation to engage in general cognitive activity were excluded from the analyses, as our focus was specifically on activity-specific motivational dimensions. In addition to the four core MCAQ dimensions (perceived capacity, intrinsic motivation, identified motivation, and external motivation), we added one item measuring amotivation for each cognitive activity. The amotivation item was phrased as: “I don’t know why I do it/I do it for no particular reason”.^1^ This addition brought the total number of administered items to 60. For each cognitive activity, examples were provided to help respondents better understand the scope of the activity (e.g., “Analyze and solve problems, such as comparing prices, adapting to an unexpected situation, or resolving a conflict”).

### 3.2 Results

#### 3.2.1 Parallel analysis

First, we conducted a parallel analysis (Horn, 1965) using O’Connor’s (2000) SPSS syntax. Parallel analysis is a statistical technique used to decide how many components to retain in an EFA. This analysis generates eigenvalues from the raw data, along with 95th percentile eigenvalues derived from a Monte Carlo simulation. The results of the analysis indicated that one single factor contained the perceived capacity items, whereas two factors emerged for intrinsic motivation, identified motivation, and external motivation, respectively (Table 1).

**Table 1:** Results of parallel analysis, Study 1.

Factor	Eigenvalue from perceived capacity	Eigenvalue from intrinsic motivation	Eigenvalue from identified motivation	Eigenvalue from external motivation	Mean eigenvalue from Monte Carlo simulation	95th percentile eigenvalue from Monte Carlo simulation
1	4.417	3.571	3.853	4.839	1.254	1.321
2	1.186	1.344	1.373	1.321	1.188	1.237
3	1.056	1.107	1.078	1.010	1.139	1.179
4	0.936	0.974	0.892	0.832	1.097	1.126
5	0.797	0.804	0.833	0.628	1.052	1.087
6	0.708	0.766	0.711	0.574	1.0123	1.037
7	0.630	0.732	0.671	0.553	0.976	1.004
8	0.552	0.666	0.634	0.509	0.939	0.970
9	0.479	0.614	0.594	0.492	0.899	0.940
10	0.461	0.522	0.505	0.469	0.860	0.896
11	0.430	0.504	0.451	0.395	0.815	0.850
12	0.348	0.396	0.405	0.378	0.769	0.817

Where eigenvalues of factors from the actual data (column 2–5) exceed the 95th percentile eigenvalues from the Monte Carlo simulation (column 7), those factors can be considered actual factors present in the dataset.

#### 3.2.2 Exploratory factor analysis

To further investigate the bi-factorial structure within the dimensions of intrinsic, identified and external motivation, we conducted an additional exploratory factor analysis using Unweighted Least Squares (ULS) extraction with Promax rotation, specifying a two-factor solution for each dimension (Table 2). This analysis revealed that some activities loaded strongly onto the first factor, while others loaded onto the second factor or both. To optimize the factorial structure, we excluded cognitive activities that exclusively loaded on the second factor (e.g., “do mind games”) and retained those that primarily loaded on the first factor (e.g., “*memorize*,” “*calculate*”). The activities “*read*,” “*write*,” “*learn*,” and “*create*” showed ambiguous results with loadings on both factors. This may be because their accompanying examples were not sufficiently framed as describing cognitive activities, but rather entertainment activities (e.g., “*read magazines*”), which could artificially enhance intrinsic motivation. Consequently, we decided to exclude the activities “*create*,” “*write*,” and “*learn*,” and to retain the activity “*read*” by refining the examples provided. Additionally, although the activity “*succeed in mental ability tests*” loaded more strongly on the second factor, we opted to retain it due to its practical importance in evaluating cognitive abilities in older adults.

**Table 2:** Factor analysis results of the motivation dimensions, Study 1.

Everyday cognitive activities	Pattern matrix					
	Intrinsic motivation		Identified motivation		External motivation	
	Factor		Factor		Factor	
	1	2	1	2	1	2
To read		0.48	0.40			
To write	0.33	0.30	0.58			0.63
To calculate	0.36		0.58		0.35	0.34
To learn		0.37	0.59		0.33	0.35
To do mind games		0.66		0.69		0.55
To analyze and solve problems	0.60		0.67			0.67
To use your sense of direction	0.68		0.51		0.68	
To memorize	0.62		0.55		0.68	
To remember	0.68		0.57		0.77	
To organize	0.35		0.54		0.79	
To create				0.34	0.72	
To succeed in mental abilities tests		0.42		0.60	0.32	

Extraction method was unweighted least squares.

These decisions enabled us to maintain a theoretically coherent four-factor structure, representing the three motivational dimensions of self-determination theory alongside the perceived capacity dimension. The final version of the MCAQ retains eight cognitive activities, specifically: (1) “*Analyze and solve problems*”, (2) *“Read short texts*”, (3) “*Do calculations in your head*”, (4) “*Use your sense of direction*”, (5) “*Memorize things*”, (6) “*Remember things*”, (7) “*Plan or organize tasks*”, and (8) “*Succeed on mental ability tests*”.

### 3.3 Discussion

Exploratory factor analyses revealed the presence of a distinct perceived capacity dimension, along with two sub-dimensions for each motivational construct. To ensure each motivational dimension loaded only on one factor, we selected eight cognitive activities, leading to a final four-factor structure. This solution aligns coherently with the theoretical frameworks guiding the scale’s development and provides a coherent representation of the motivational dimensions.

## 4 Study 2

The objective of Study 2 was to validate the factorial structure of the MCAQ identified in Study 1 using confirmatory factor analysis (CFA). Building on the exploratory factor analyses conducted previously, Study 2 aimed to test whether the 4-dimension structure could be confirmed in a new sample of French-speaking participants. Specifically, we evaluated a model comprising four latent variables (i.e., perceived capacity, intrinsic motivation, identified motivation, and external motivation) each represented by the items retained from Study 1. In addition, we examined the internal consistency of each dimension. This study thus provides a more rigorous test of the MCAQ’s factor structure and sets the stage for subsequent investigations of its convergent and predictive validity.

### 4.1 Methods

#### 4.1.1 Participants and procedure

A total of 520 French-speaking participants were recruited through the panel research platform FouleFactory. Participants included 275 women (52.9%) and 245 men (47.1%). Ages ranged from 18 to 76 years (*M* = 37.83, *SD* = 11.09). Regarding educational attainment, 7.7% had completed secondary education or less, 13.8% had obtained a baccalaureate or vocational baccalaureate, 46.5% had completed 2–3 years of higher education, and 31.9% had completed five or more years of higher education. Regarding household income, 31.6% reported an annual pre-tax household income below €20,000, 43.5% between €20,000 and €40,000, and 24.9% above €40,000. The study followed the same procedure as in Study 1, with participants completing the MCAQ items online after providing informed consent.

#### 4.1.2 Measures

The MCAQ was administered as described in previous studies, targeting the eight cognitive activities identified in Study 1. In total, participants responded to 32 items, distributed across the four dimensions. These items were designed to capture participants’ perceived capacity and levels of intrinsic, identified, and external motivation for engaging in each cognitive activity.

### 4.2 Results

#### 4.2.1 Internal consistency

The internal consistency of each dimension was found to be acceptable, with Cronbach’s alphas as follows: perceived capacity (α = 0.73), intrinsic motivation (α = 0.75), identified motivation (α = 0.79), and external motivation (α = 0.87).

#### 4.2.2 Confirmatory factor analysis

The CFA tested a model comprising four latent constructs, each represented by eight observed indicators. To account for shared variance across cognitive activities, we correlated the residuals of observed indicators between latent variables (Cole et al., 2007). Missing data were handled using maximum likelihood estimation. Results indicated a good fit to the data, χ^2^(410) = 1157.995, *p* < 0.05; RMSEA = 0.059; CFI = 0.899.^2^ Table 3 provides the CFA standardized and unstandardized coefficients.

**Table 3:** Standardized and unstandardized coefficients, Study 2.

Observed indicators	Latent factor	*β*	*B*	*SE*
Perceived capacity—Solve problems	Perceived capacity	0.65	1.00	
Perceived capacity—Calculate	Perceived capacity	0.53	1.22	0.11
Perceived capacity—Sense of direction	Perceived capacity	0.41	1.00	0.11
Perceived capacity—Memorize	Perceived capacity	0.56	1.23	0.11
Perceived capacity—Remember	Perceived capacity	0.53	1.13	0.11
Perceived capacity—Organize	Perceived capacity	0.44	0.83	0.09
Perceived capacity—Succeed in tests	Perceived capacity	0.55	1.00	0.09
Perceived capacity—Read	Perceived capacity	0.49	0.78	0.08
Intrinsic—Solve problems	Intrinsic motivation	0.48	1.00	
Intrinsic—Calculate	Intrinsic motivation	0.49	1.21	0.13
Intrinsic—Sense of direction	Intrinsic motivation	0.49	1.27	0.14
Intrinsic—Memorize	Intrinsic motivation	0.72	1.66	0.15
Intrinsic—Remember	Intrinsic motivation	0.62	1.40	0.14
Intrinsic—Organize	Intrinsic motivation	0.43	0.94	0.11
Intrinsic—Succeed in tests	Intrinsic motivation	0.35	0.82	0.11
Intrinsic—Read	Intrinsic motivation	0.59	1.29	0.13
Identified—Solve problems	Identified motivation	0.59	1.00	
Identified—Calculate	Identified motivation	0.62	1.22	0.10
Identified—Sense of direction	Identified motivation	0.64	1.32	0.11
Identified—Memorize	Identified motivation	0.71	1.27	0.10
Identified—Remember	Identified motivation	0.64	1.17	0.10
Identified—Organize	Identified motivation	0.53	0.92	0.08
Identified—Succeed in tests	Identified motivation	0.29	0.62	0.10
Identified—Read	Identified motivation	0.59	0.92	0.08
External—Solve problems	External motivation	0.66	1.00	
External—Calculate	External motivation	0.64	1.03	0.07
External—Sense of direction	External motivation	0.62	0.99	0.08
External—Memorize	External motivation	0.77	1.17	0.07
External—Remember	External motivation	0.75	1.17	0.08
External—Organize	External motivation	0.69	1.11	0.08
External—Succeed in tests	External motivation	0.48	0.76	0.07
External—Read	External motivation	0.70	1.23	0.09

### 4.3 Discussion

The CFA results provided empirical support for the four-factor structure of the MCAQ, encompassing perceived capacity, intrinsic motivation, identified motivation, and external motivation. Importantly, this structure replicated the four-factor solution identified through the EFA in Study 1, providing convergent evidence for the proposed factorial organization of the scale in an independent sample. Additionally, each dimension demonstrated acceptable internal consistency.

## 5 Study 3

Building on the factor structure established and confirmed in Studies 1 and 2, Study 3 aimed to further evaluate the MCAQ in both its original French version and a newly translated English version. The primary goals were to replicate and extend the factorial analyses in new samples of French- and English-speaking participants and to establish the external validity of both versions. Convergent validity was assessed by examining correlations with related constructs, including the Need for Cognition scale, the Global Motivation Scale, the Personality in Intellectual Aging Context Inventory, and the Metamemory in Adulthood scale. Predictive validity was examined through relationships with self-reported engagement in cognitive tasks, providing insight into the MCAQ’s ability to predict real-world cognitive behavior. Finally, to assess the stability of the instrument over time, we evaluated test–retest reliability by correlating responses from the initial administration (Time 1) with those collected 2 months later (Time 2). Together, this study provides a critical test of both the cross-linguistic applicability and the external validity of the MCAQ, building on the structural validation conducted in earlier studies.

### 5.1 Methods

#### 5.1.1 Participants and procedure

We recruited 260 English-speaking participants via Amazon Mechanical Turk and 255 French-speaking participants via FouleFactory. They were all compensated financially for their participation. Seven participants from each language group were excluded for failing to complete the questionnaire fully. An additional twelve English-speaking and thirteen French-speaking participants were excluded for failing to answer the attention check questions correctly. We also screened for participants who completed the questionnaire too quickly (i.e., in less than 10 min), as rapid responses are often associated with straight-lining behavior (Zhang and Conrad, 2014). Following this screening process, we retained 205 English-speaking participants (ages 22–74; *M* = 40.77, *SD* = 11.92) and 231 French-speaking participants (ages 19–75; *M* = 41.54, *SD* = 12.52). Among the participants who reported their gender, 258 were women (59.3%) and 177 were men (40.7%). Regarding educational attainment, 17.0% had completed secondary education or less, 24.6% had obtained a baccalaureate or vocational baccalaureate, 40.9% had completed 2–3 years of higher education, and 17.5% had completed five or more years of higher education. For the test–retest reliability analysis, 304 participants completed the MCAQ a second time: 128 English-speaking and 176 French-speaking.

#### 5.1.2 French-English back translation

The MCAQ was translated into English following the International Test Commission’s guidelines for test translation (van de Vijver and Hambleton, 1996), employing a forward-backward translation process and a committee approach (Gudmundsson, 2009; Peters and Passchier, 2006). Initially, a native English speaker familiar with the research area translated the MCAQ from French into English. The translation was reviewed, and any unclear expressions were resolved. Subsequently, a native French-speaking translator performed a back-translation from English into French. Discrepancies between the original and back-translated versions were discussed until consensus was reached, ensuring linguistic and conceptual equivalence.

#### 5.1.3 Measures

##### 5.1.3.1 MCAQ

The MCAQ was administered as in Study 2, including the four items assessing motivation to engage in general cognitive activities.

##### 5.1.3.2 Need for cognition (NFC)

We adapted a short version of the NFC scale (Cacioppo et al., 1984) consisting of 11 items, with responses measured on a 4-point Likert scale. Higher scores indicated a stronger need for cognition.

##### 5.1.3.3 Global Motivation Scale (GMS)

The GMS (Guay et al., 2003) assesses various types of intrinsic motivation (knowledge, stimulation, and accomplishment) and extrinsic motivation (identified, introjected, and external), along with amotivation. Each subscale contains four items, and participants respond using a 7-point Likert scale. Only intrinsic and extrinsic motivation were measured, a-motivation was excluded. Higher scores indicated stronger motivation levels.

##### 5.1.3.4 Personality in intellectual aging context (PIC)

We used two subscales from the PIC (Lachman et al., 1982): internal control (perceived intellectual competence and control over one’s intellectual functioning) and achievement motivation (desire to maintain intellectual functioning). Each subscale contains 12 items, measured on a 6-point Likert scale. Higher scores were indicative of stronger control and motivation.

##### 5.1.3.5 Metamemory in Adulthood (MIA)

Given that the MIA was primarily included due to its theoretical overlap with the perceived capacity dimension, we only included two of its dimensions (Boucheron, 1995; Dixon et al., 1988): the capacity dimension assesses perceived memory capacity in everyday situations (17 items) and the achievement dimension measures motivation to perform well in memory tasks (16 items). Responses were given on a 5-point scale. Higher scores indicated higher perceived ability or motivation.

##### 5.1.3.6 Engagement in cognitive tasks

We developed six items to assess individuals’ engagement in cognitive tasks, including three reverse-scored items. Sample items included: “If I encounter difficulties in a task requiring concentration and thought, I increase my effort to complete it” and “If I encounter difficulties in a task requiring concentration and thought, I persist in carrying it out”. Participants rated their responses on a 7-point Likert scale, ranging from 1 (not at all true for me) to 7 (very true for me).

### 5.2 Results

#### 5.2.1 Internal consistency

The internal consistency of all MCAQ dimensions was deemed acceptable for both language groups and across both measurement occasions, with Cronbach’s α ranging from 0.74 to 0.92 (Table 4). Moreover, correlation analyses between the MCAQ dimensions revealed that intrinsic motivation was positively associated with identified motivation for both general items and specific activity dimensions. External motivation, however, showed weak correlations with intrinsic and identified motivation (Table 5). Perceived capacity was positively correlated with intrinsic and identified motivation but exhibited either no or negative correlations with external motivation. These findings are consistent with the predictions of self-determination theory (Chemolli and Gagné, 2014; Ryan and Deci, 2000).

**Table 4:** Cronbach’s α (English/French speaking participants) at Time 1 and 2, Study 3.

Motivation dimension	Cronbach’s *α*	
	Time 1	Time 2
Perceived capacity	0.84/0.77	0.84/0.81
Intrinsic motivation	0.86/0.74	0.88/0.82
Identified motivation	0.86/0.78	0.88/0.82
External motivation	0.92/0.89	0.94/0.91

**Table 5:** Correlations (English/French speaking participants) between the MCAQ dimensions, general items, and activity-specific dimensions, Study 3.

MCAQ section	Motivation dimension	1	2	3	4	5	6	7
General items	1. Perceived capacity	1						
	2. Intrinsic motivation	0.52**/0.48**	1					
	3. Identified motivation	0.49**/0.38**	0.55**/0.66**	1				
	4. External motivation	−0.17*/0.02	−0.33**/−0.26**	−0.22**/0.12^†^	1			
Activity-specific dimensions	5. Perceived capacity	–	0.50**/0.53**	0.60**/0.48**	−0.18**/0.05	1		
	6. Intrinsic motivation	0.47**/0.36**	0.66**/0.56**	0.48**/0.48**	−0.23**/−0.11	0.62**/0.53**	1	
	7. Identified motivation	0.47**/0.33**	0.44**/0.47**	0.66**/0.63**	−0.15*/−0.06	0.60**/0.52**	0.49**/0.64**	1
	8. External motivation	−0.04/0.00	−0.11/−0.13	−0.04/−0.04	0.70**/0.72**	−0.03/0.07	−0.11/−0.22**	0.05/−0.01

^†^*p* < 0.10, *p < 0.05. **p < 0.01.

#### 5.2.2 Test–retest reliability

Correlations between Time 1 and Time 2 for the general items were all significant and ranged from 0.37 to 0.58 (see Supplementary Table S1). Correlations between the subscales scores were also significant and ranged from 0.49 to 0.73, indicating satisfactory test–retest reliability of the MCAQ.

#### 5.2.3 Confirmatory factor analysis

Separate CFAs were conducted for English-speaking and French-speaking participants using the model estimated in Study 2. Both analyses demonstrated adequate fit to the data among English-speaking participants, χ^2^(410) = 813.441, *p* < 0.05; RMSEA = 0.069; CFI = 0.887, and French-speaking participants, χ^2^(410) = 721.283, *p* < 0.05; RMSEA = 0.058; CFI = 0.917. Multigroup CFAs further indicated that the MCAQ factor structure is invariant across languages (details are provided in the Supplementary material).

#### 5.2.4 Convergent validity

Convergent validity was supported by positive correlations between the MCAQ dimensions and related measures (Table 6). Specifically, in both language groups, the NFC scale showed a positive correlation with the MCAQ dimension of intrinsic motivation, while a negative correlation with external motivation. Additionally, significant correlations were found between the MCAQ dimensions and corresponding dimensions of the GMS, such as the positive correlation between MCAQ intrinsic motivation and GMS intrinsic motivation related to knowledge. The MCAQ dimensions that oppose GMS dimensions according to the self-determination continuum were negatively correlated (e.g., MCAQ external motivation negatively correlated with GMS intrinsic motivation). Positive correlations were also observed between indicators of perceived capacity from the MCAQ and from the PIC (i.e., internal control subscale) and the MIA (i.e., capacity subscale), as well as between indicators of achievement motivation from the PIC (i.e., achievement motivation dimension) or the MIA (i.e., achievement subscale) and the intrinsic and identified motivation dimensions of the MCAQ, across both language groups.

**Table 6:** Correlations (English/French speaking participants) between the MCAQ and other measures, Study 3.

MCAQ section	Motivation dimension	NFC	GMS						PIC		MIA		Engagement in cognitive tasks
			Knowledge	Accomplishment	Stimulation	Identified	Introjected	External	Internal control	Achievement motivation	Capacity	Achievement	
General items	Perceived capacity	0.44**/0.35**	0.28**/0.20**	0.24**/0.10	0.22**/0.11^†^	0.31**/0.20**	−0.17*/0.03	−0.05/0.01	0.38**/0.20**	0.41**/0.19**	0.35**/0.32**	0.26**/0.07	0.50**/0.32**
	Intrinsic motivation	0.64**/0.50**	0.51**/0.41**	0.43**/0.29**	0.38**/0.25**	0.42**/0.27**	−0.22**/0.03	−0.02/0.02	0.41**/0.42**	0.60**/0.45**	0.29**/0.26**	0.31**/0.25**	0.57**/0.45**
	Identified motivation	0.49**/0.30**	0.39**/0.39**	0.30**/0.33**	0.25**/19**	0.47**/0.38**	−0.02/0.03	0.05/0.07	0.49**/0.41**	0.51**/0.41**	0.29**/0.18**	0.30**/0.27**	0.43**/0.32**
	External motivation	−0.35**/−0.12^†^	−0.21**/−0.13*	−0.11/−0.04	−0.13/−0.09	−0.12/−0.00	0.32**/0.28**	0.21**/0.25**	−0.26**/−0.12^†^	−0.28**/−0.14**	−0.21**/−0.13^†^	−0.16*/−0.10	−0.30**/−0.15**
Activity-specific dimensions	Perceived capacity	0.44**/0.32**	0.41**/0.28**	0.30**/0.25**	0.28**/0.16*	0.43**/0.26**	−0.09/0.09	0.03/0.06	0.55**/0.35**	0.52**/0.28**	0.54**/0.45**	0.40**/0.16*	0.49**/0.40**
	Intrinsic motivation	0.55**/0.35**	0.50**/0.42**	0.47**/0.28**	0.38**/0.29**	0.43**/0.27**	−0.07/0.02	0.09/0.05	0.39**/0.49**	0.59**/0.48**	0.36**/0.34**	0.34**/0.27**	0.39**/0.35**
	Identified motivation	0.44**/0.24**	0.38**/0.40**	0.28**/0.27**	0.24**/0.24**	0.48**/0.37**	−0.03/−0.01	0.03/0.01	0.57**/0.46**	0.52**/0.50**	0.32**/0.25**	0.38**/0.28**	0.47**/0.33**
	External motivation	−0.23**/−0.15*	−0.20**/0.07	−0.07/0.03	−0.10/−0.05	0.00/0.13^†^	0.31**/0.32**	0.23**/0.35**	−0.12^†^/−0.18**	−0.13^†^/−0.17*	−0.16*/−0.13^†^	−0.03/−0.09	−0.16*/−0.16*

^†^*p* < 0.10, *p < 0.05. **p < 0.01.

#### 5.2.5 Predictive validity

Consistent with expectancy-value theory and self-determination theory, engagement in behavioral tasks was positively correlated with perceived capacity, intrinsic motivation, and identified motivation, while negatively correlated with external motivation (Table 6).

### 5.3 Discussion

Study 3 further validated the MCAQ in both French and English, confirming its internal reliability and factorial structure. The scale demonstrated satisfactory convergent validity through high correlations with similar measures and acceptable predictive validity with behavioral task engagement. These results support the robustness and applicability of the MCAQ across different languages and contexts.

## 6 Study 4

Study 4 aimed to extend the validation of the MCAQ by examining its factor structure and predictive validity in a large, real-world educational context. While Study 3 focused on self-reported engagement in cognitive activities, Study 4 sought to determine whether MCAQ dimensions predict actual performance outcomes. To achieve this, we leveraged data from the ProFAN study, a nationwide longitudinal project initiated in 2017 by the French Ministry of Education, which includes over 10,000 students enrolled in vocational programs. The ProFAN study was designed to evaluate the impact of cooperative learning on a diverse sample of high-school students across numerous vocational schools in France. The school environment, with its emphasis on educational performance, provides an optimal context and an ecologically valid setting to evaluate the MCAQ. Using this extensive dataset, Study 4 not only examines the factor structure of the MCAQ in a large and diverse student sample but also investigates its ability to predict meaningful performance outcomes, thereby extending the generalizability and applied relevance of the scale established in previous studies.

### 6.1 Methods

#### 6.1.1 Participants and procedure

The ProFAN project was conducted across two cohorts of students from 108 vocational schools in France, with data collected in 2017–2018 (Cohort 1) and 2018–2019 (Cohort 2). Each cohort was assessed at two time points, 1 year apart. Cohort 1 included 4,620 students at Time 1 (Men: *n* = 2,054; Women: *n* = 2,544; Age: *M* = 16.47, *SD* = 0.88) and 3,899 students at Time 2. Cohort 2 comprised 4,436 students at Time 1 (Men: *n* = 1,904; Women: *n* = 2,497; Age: *M* = 16.41, *SD* = 0.85) and 3,930 students at Time 2.

Participating schools were selected by the French Ministry of Education and participated voluntarily. Within each school, classes were randomly selected to ensure a representative sample. Student participation was mandatory as the study was integrated into their regular curriculum. Data collection occurred during normal school hours using the school’s computers. Students were required to complete an online questionnaire via the online ProFAN platform, which included the MCAQ among other measures (for a full description of the ProFAN research, see https://e-fran.education.gouv.fr/profan/). They were allotted 1 h to complete the questionnaire.

#### 6.1.2 Measures

##### 6.1.2.1 MCAQ

The MCAQ was administered in French at both time points for each cohort. The measure included the same items as detailed in previous studies.

##### 6.1.2.2 Cognitive performance

We operationalized cognitive performance through self-reported grades in mathematics and French. Indeed, students’ grades may serve as reliable proxies for assessing academic competencies and performance (see Mella et al., 2021). More specifically, students were asked to report their grades from the year preceding data collection. Because grades were self-reported, they may be subject to recall and social-desirability biases and should therefore be interpreted as indicators of perceived academic performance rather than objective measures of cognitive performance. Although the reported grades were from an earlier period, this should not undermine the predictive power of the MCAQ, as high school students’ grades are known to be highly stable throughout their academic trajectory (Guskey, 2011). In the French education system, grades are given on a 20-point scale. To compensate for errors when reporting their grades, we converted students’ self-reported grades to a five-point scale (1 = 0–4.9; 2 = 5–8.9; 3 = 9–12.9; 4 = 13–15.9; and 5 = 16–20) and averaged them into a single performance indicator. Note however that grades were collected only at Time 1 for both cohorts, and not at Time 2.

##### 6.1.2.3 Socio-demographics

Several socio-demographic variables were included as control variables in the analyses: student gender (0 = female, 1 = male), age, and parents’ socio-economic status.

### 6.2 Results

#### 6.2.1 Internal consistency

Cronbach’s alphas for the MCAQ dimensions ranged from 0.78 to 0.89, demonstrating satisfactory internal consistency across both time points and cohorts. Furthermore, correlation analyses revealed positive relationships between all dimensions of the scale, including both general items and domain-specific subscales, across both assessment times and cohorts (Table 7). Contrary to our hypotheses, external regulation exhibited positive correlations, albeit small (rs ≈ 0.20), with the other dimensions of the scale. Given the large sample size, these small associations may have reached statistical significance despite their limited magnitude. Accordingly, although the correlations were statistically significant, their small effect sizes suggest that they should be interpreted cautiously and may have limited substantive significance.

**Table 7:** Correlations between the MCAQ dimensions across time and cohorts, Study 4.

MCAQ section	Motivation dimension	Time 1				Time 2			
		1	2	3	4	5	6	7	8
General items	1. Perceived capacity								
	Cohort 1	1				1			
	Cohort 2								
	2. Intrinsic motivation								
	Cohort 1	0.41***	1			0.45***	1		
	Cohort 2	0.41***				0.43***			
	3. Identified motivation								
	Cohort 1	0.40***	0.68***	1		0.44***	0.68***	1	
	Cohort 2	0.40***	0.68***			0.43***	0.69***		
	4. External motivation								
	Cohort 1	0.15***	0.18***	0.29***	1	0.14***	0.18***	0.29***	1
	Cohort 2	0.15***	0.18***	0.29***		0.17***	0.22***	0.33***	
Activity-specific dimensions	5. Perceived capacity								
	Cohort 1	1				1			
	Cohort 2								
	6. Intrinsic motivation								
	Cohort 1	0.48***	1			0.48***	1		
	Cohort 2	0.46***				0.54***			
	7. Identified motivation								
	Cohort 1	0.49***	0.70***	1		0.56***	0.75***	1	
	Cohort 2	0.49***	0.73***			0.56***	0.79***		
	8. External motivation								
	Cohort 1	0.13***	0.25***	0.36***	1	0.17***	0.31***	0.39***	1
	Cohort 2	0.20***	0.36***	0.45***		0.24***	0.37***	0.43***	

***p < 0.001.

#### 6.2.2 Test–retest reliability

Test–retest reliability was evaluated through correlation analyses and paired t-tests comparing scores from Time 1 and Time 2 within both cohorts (see Supplementary Table S2). Correlations for the general items and subscales ranged from 0.17 to 0.53, indicating low to moderate test–retest reliability over the one-year interval, depending on the dimension. We additionally examined mean-level changes over time to get an indication of temporal stability. Almost all Time 1-Time 2 differences were statistically significant, which may be influenced by the large sample sizes. To address this, we calculated effect sizes using Hedges’ g, with values ranging from 0.01 to 0.17. These small effect sizes suggest minimal Time 1-Time 2 differences.

#### 6.2.3 Confirmatory factor analysis

CFAs were conducted to validate the MCAQ’s factor structure across time and within both cohorts. The specified model included four latent factors, each represented by eight observed indicators. As in previous studies, residuals of observed indicators across latent factors were correlated. For Time 1, the model demonstrated an excellent fit to the data: χ^2^(876) = 8665.736, *p* < 0.001; RMSEA = 0.044; CFI = 0.936. Similar results were obtained for Time 2, χ^2^(876) = 8390.370, *p* < 0.001; RMSEA = 0.047; CFI = 0.942. All observed indicators had statistically significant factor loadings (*p* < 0.001), with parameter estimates exceeding 0.51, indicating accurate factor assignments.

#### 6.2.4 Predictive validity

To assess predictive validity, we separately regressed students’ grades on the general MCAQ items and activity-specific dimensions (Table 8). These analyses were conducted for both cohorts and controlled for students’ age, gender, and parents’ socio-economic status. Since self-reported grades were only measured at Time 1, the relationship between the MCAQ and performance was not examined at Time 2. As expected, we found consistent evidence that perceived capacity and identified motivation positively predicted performance in both cohorts and for both the general items and activity-specific dimensions. Intrinsic motivation was positively linked to performance only through the general item, not the subscales, and in only one cohort. External regulation was negatively related to performance, but this was observed in only one cohort and only with the subscale, not the general item.

**Table 8:** Multiple regressions examining the relationship between the MCAQ and cognitive performance, Study 4.

MCAQ section	Motivation dimension	*β*	*p*	95% CI
General item	Perceived capacity			
	Cohort 1	0.26	<0.001	[0.223, 0.298]
	Cohort 2	0.25	<0.001	[0.218, 0.289]
	Intrinsic motivation			
	Cohort 1	−0.01	0.640	[−0.059, 0.036]
	Cohort 2	0.05	0.029	[0.005, 0.096]
	Identified regulation			
	Cohort 1	0.07	0.007	[0.018, 0.113]
	Cohort 2	0.06	0.010	[0.014, 0.107]
	External regulation			
	Cohort 1	−0.02	0.362	[−0.052, 0.019]
	Cohort 2	−0.01	0.894	[−0.037, 0.032]
Activity-specific dimensions	Perceived capacity			
	Cohort 1	0.18	<0.001	[0.135, 0.219]
	Cohort 2	0.21	<0.001	[0.167, 0.243]
	Intrinsic motivation			
	Cohort 1	0.05	0.059	[−0.002, 0.102]
	Cohort 2	0.02	0.467	[−0.032, 0.070]
	Identified regulation			
	Cohort 1	0.08	0.005	[0.024, 0.131]
	Cohort 2	0.11	<0.001	[0.058, 0.164]
	External regulation			
	Cohort 1	−0.03	0.125	[−0.067, 0.008]
	Cohort 2	−0.04	0.022	[−0.081, −0.006]

### 6.3 Discussion

Study 4 gave support for internal consistency, and overall validity of the MCAQ. The large-scale and longitudinal data from the ProFAN study, encompassing two distinct cohorts over a two-year period, supported the factor structure of the MCAQ. Test–retest reliability (over a one-year interval) was found to be small to moderate, depending on the dimensions. Thes results should be interpreted in the context of the substantially longer retest interval and the adolescent sample, for whom motivational orientations and perceived capacities may legitimately evolve over the course of a year.

Additionally, the MCAQ showed partial evidence of predictive validity. Perceived capacity and identified motivation were positively and consistently associated with students’ performance. Although intrinsic and extrinsic motivation were associated with performance in the expected directions, these relationships were less consistent across cohorts and across general versus activity-specific measures of the MCAQ. One possible explanation for this variability is that the relationship between motivation and performance may be context dependent. In particular, performance in the present study was assessed in an academic context. While students may be intrinsically motivated for everyday cognitive activities, their academic engagement may rather be driven by the perceived utility and importance of academic achievement for future goals (Burton et al., 2006). They primarily engage in academic activities because they recognize their importance for achieving academic or professional goals, even when these activities are not intrinsically enjoyable. Conversely, being intrinsically motivated toward everyday cognitive activities does not necessarily imply greater motivation to perform specific academic tasks. Similarly, low levels of extrinsic motivation for cognitive activities may not translate into lower academic performance. Thus, the fact that the associations between intrinsic and external motivation and performance were generally in the expected direction but varied in statistical significance across cohorts and MCAQ scores, may reflect differences in the extent to which general motivational dispositions translate into motivation in the specific academic context in which performance was assessed. Future research could examine this possibility by assessing motivation and performance within the same domain and at comparable levels of specificity.

## 7 General discussion

The present research aimed to construct and validate the MCAQ, a novel assessment tool designed to evaluate individuals’ motivation to engage in various cognitive activities. Grounded in expectancy-value theory (Eccles and Wigfield, 2020, 2024) and self-determination theory (Ryan and Deci, 2000, 2020), we established a factor structure that accounts for the multidimensional nature of motivation. The final version of the MCAQ comprises 32 items, organized around four dimensions: perceived capacity, intrinsic motivation, identified motivation, and external motivation. Eight everyday cognitive activities were selected (i.e., “*Analyze and solve problems*”, “*Read short text*”, “*Perform mental calculations*”, “*Use your sense of direction*”, “*Memorize things*”, “*Remember things*”, “*Plan or organize tasks*”, and “*Succeed on mental ability tests*”). In addition, the MCAQ includes a brief four-item scale assessing general motivation to engage in cognitive effort. These items may be administered either alongside or independently of the activity-specific items. Participants rate their perceived capacity, intrinsic motivation, identified motivation, and external motivation to “use their brain for activities that require concentration and reflection”.

Across four studies, the results of the present research provided satisfactory psychometric evidence for the validity of the MCAQ in both English and French. Each of the four dimensions demonstrated good internal consistency, with Cronbach’s α coefficients ranging from 0.74 to 0.94. Test–retest reliability analyses conducted in Studies 3 and 4 generally supported the reliability of the MCAQ across repeated assessments, although the magnitude of the coefficients varied depending on the retest interval and the dimensions assessed. Exploratory and confirmatory factor analyses, conducted across four studies, consistently corroborated the validity of the scale’s factor structure. The MCAQ also exhibited good construct validity, with correlational analyses revealing coherent patterns of relationships among the MCAQ subscales. Furthermore, Study 3 demonstrated adequate convergent validity for the MCAQ, as it correlated positively with established measures of motivation for cognitive activites, including the Need for Cognition scale, the Global Motivation Scale, the Personality in Intellectual Aging Context scale, and the Metamemory in Adulthood scale.

Additionally, our results provided mixed evidence for the predictive validity of the MCAQ. Study 3 revealed the expected associations between the MCAQ dimensions and self-reported engagement in cognitive tasks, providing initial evidence that the measure is related to individuals’ engagement in cognitive activities. Study 4 further examined whether MCAQ scores were associated with academic performance in an educational context, using longitudinal data from the ProFAN study, which includes a sample of over 10,000 vocational students in France. Several dimensions of the MCAQ were associated with students’ performance, assessed through self-reported grades in French and mathematics, although these associations were not fully consistent across cohorts and MCAQ measures. We notably found adequate relationships between perceived capacity, identified motivation, and student performance. These findings aligned with the motivational theories underpinning the MCAQ, which predict that the perception of being capable of engaging in a specific task enhances actual task performance (Bandura, 1993; Eccles and Wigfield, 2020). Additionally, undertaking a particular action while being motivated by how it can bring personal benefits reinforces good performance in executing that action (Ryan et al., 2022). Intrinsic and external motivation, however, were less consistently associated with performance, which may be attributed to the specificity of the school context that may not imply such motivations as central drivers of academic achievement (notably in comparison with identified motivation).

Consequently, the analyses conducted across the current studies attest to the validity of the scale in measuring motivation for cognitive activities.

### 7.1 Theoretical and practical contributions

The MCAQ offers significant contributions. While existing scales measuring motivation for engaging in cognitive activities have evident limitations, such as focusing on specific populations (e.g., older adults, college students), particular cognitive domains (e.g., creativity, learning), or only one facet of motivation (e.g., intrinsic motivation), the MCAQ represents a substantial advancement. It provides an integrative instrument for evaluating motivation for cognitive activities beyond the specifics of a particular context, population, or cognitive activity. In addition, the fine distinction between four dimensions of motivation, reflecting dominant theoretical conceptualizations in motivation research (Eccles and Wigfield, 2024; Ryan and Deci, 2020), can contribute to a better understanding of the psychological mechanisms underlying engagement in tasks that involve everyday cognitive activities. For example, the MCAQ could be used to investigate how different dimensions of motivation predict academic performance across various educational levels and disciplines.

Moreover, the development of general items can be useful for researchers who wish to assess motivation for engaging in cognitive activities using a short scale, for instance in large-scale surveys. It is also important to note that the MCAQ has been validated in two languages, French and English, which underscores its potential applicability across diverse linguistic contexts and makes it a potentially useful scale for international research aiming at exploring the role of motivation in cognitive performance. Future studies should nevertheless examine its cross-cultural validity, as cross-language validation does not necessarily imply cross-cultural equivalence.

Importantly, the MCAQ can be particularly useful in practical settings, especially for practitioners interested in the development and maintenance of cognitive abilities. For instance, distinguishing changes in cognitive abilities from changes in motivation for using cognitive abilities would be crucial for a better understanding of cognitive decline and, consequently, for developing appropriate remediation strategies. Additionally, such an enhanced understanding can inform the creation of targeted interventions and educational strategies aimed at fostering motivation for cognitive activities and improving cognitive performance outcomes, for example among aging populations, students, or individuals with cognitive impairment. Thus, the MCAQ is poised to become a useful resource for practitioners and educators seeking to understand and promote motivation for cognitive activities.

## 8 Limitations

While the present studies offered compelling support for the psychometric properties of the MCAQ, several limitations warrant attention. Firstly, the preliminary qualitative phase used to generate the initial pool of cognitive activities involved a small convenience sample. Although this phase was intended to support item generation rather than to obtain a representative description of everyday cognitive activities, future research could examine whether the activity categories identified here are reproduced across larger, more diverse, and culturally varied samples.

Secondly, the factor analyses conducted in Study 1 led us to exclude three cognitive activities (“*create*,” “*write*,” and “*learn*”). Indeed, the analyses yielded ambiguous results, potentially explained by the fact that the accompanying examples reflected more entertainment-oriented activities rather than purely cognitive ones. Additionally, the activities “*create*” and “*learn*” were relatively abstract and may have been difficult for participants to evaluate over the multiple dimensions of motivation. Therefore, we concluded that these activities should not be included in the final version of the MCAQ. As a result, some domains of everyday cognitive activity are less represented in the final scale than initially intended. However, our objective was not to develop an exhaustive inventory of all possible cognitive activities, but rather to identify a set of common everyday activities that could adequately capture individual differences while maintaining a questionnaire of manageable length.

Thirdly, although the current research provided evidence for the convergent and predictive validity of the MCAQ, we did not assess its discriminant validity. This means that we did not examine whether the MCAQ is distinct from unrelated constructs. The lack of examination of discriminant validity leaves open the possibility that the MCAQ may overlap with other psychological constructs, potentially confounding the interpretation of results. Thus, it remains unclear whether the MCAQ specifically measures motivation for cognitive activities or if it captures aspects of general motivation, personality traits, or other related but distinct constructs. Future research should address this limitation by including measures of different or unrelated constructs to assess the discriminant validity of the MCAQ.

Fourthly, the MCAQ was designed to be applicable across various contexts and populations, which we corroborated in the present studies using diverse samples of participants recruited through online panel platforms. However, apart from Study 4, which specifically focused on high-school students, it is also crucial to investigate the validity of the MCAQ within specific population categories, such as older adults or individuals with cognitive impairments. This would ensure its validity and functionality for specific subgroups and potentially allow for adjustments based on the unique characteristics of each subpopulation.

Fifthly, we acknowledge that Study 4 relied on students’ self-reported grades rather than objective performance indicators. Although self-reported grades provide valuable insight into individuals’ perceptions of their academic performance, they are vulnerable to various biases, including social desirability, limited accuracy, and recall bias. Consequently, the associations observed between the MCAQ and self-reported academic performance should not be interpreted as direct evidence of predictive validity for objective cognitive performance. Future research should incorporate objective cognitive tasks and/or performance-based indicators to more rigorously assess the predictive validity of the MCAQ.

Sixthly, a limitation of the present validation concerns the model fit observed in two of the confirmatory factor analyses, where CFI values were slightly below the conventional 0.90 benchmark (0.899 and 0.887). Although the overall pattern of fit indices provided support for the hypothesized factor structure, these findings warrant caution and suggest that further studies should seek to replicate and further examine the factorial structure of the scale in novel samples.

Finally, test–retest coefficients for the MCAQ varied across the two studies. Whereas Study 3 demonstrated satisfactory short-term test–retest reliability over a two-month interval, lower correlations were observed over the one-year interval in Study 4. These findings suggest that the consistency of individual differences in MCAQ scores may be lower over longer intervals, although the lower coefficients should also be interpreted in light of the nature of the construct assessed and the characteristics of the sample. The MCAQ captures perceived capacity and motivational orientations toward everyday cognitive activities, which are likely to evolve over time, particularly during adolescence and across a one-year period. Future longitudinal studies using varying retest intervals and including diverse populations would help clarify the extent to which changes in test–retest coefficients reflect differences in measurement reliability versus genuine changes in individuals’ engagement in everyday cognitive activities.

## 9 Conclusion

The present research offered empirical evidence supporting the MCAQ as a psychometrically sound measurement tool for assessing motivation to engage in cognitive activities. This new measuring scale makes a significant contribution to the field of motivational psychology by addressing the limitations of existing measures and providing a more comprehensive and precise evaluation of motivation for engaging in cognitive activities. The MCAQ’s multidimensional structure, informed by expectancy-value theory and self-determination theory, captures the nuanced aspects of motivation and enables a fine-grained analysis of the distinct psychological mechanisms underlying cognitive engagement. This theoretically grounded approach allows for a more detailed and accurate evaluation of motivation for engaging in cognitive activities compared to existing measures. Therefore, the MCAQ represents a significant advancement by offering a valuable tool for future research and for practical applications to better promote motivation for cognitive activities within different populations and contexts.

## Data Availability

The datasets presented in this study can be found in online repositories. The names of the repository/repositories and accession number(s) can be found at: Open Science Framework: https://osf.io/terzf/?view_only=d600ecf1b345465283439c20e2bd6c15. The ProFAN data (Study 4) are available upon request from the authors.

## Footnotes

1 Although amotivation is a central construct in self-determination theory, we did not retain it in the subsequent studies because we concluded that its operationalization was not well suited to the cognitive activities assessed in the MCAQ. Specifically, the corresponding item (“*I don’t know why I do it / I do it for no particular reason*”) was difficult to interpret for activities such as analyzing and solving problems, reading, planning tasks, or recalling information, which typically involve intentional engagement. In addition, assessing amotivation for each activity substantially increased the length of the questionnaire (60 items), which was inconsistent with our aim of developing a brief and accessible measure.

2 Regarding model fit indices, commonly cited guidelines suggest that a CFI above 0.95 indicates excellent fit, whereas values above 0.90 are typically considered acceptable (McDonald and Ho, 2002). Accordingly, the CFI values observed in the present study (and another one in Study 3) were slightly below the conventional 0.90 benchmark. However, as emphasized by Marsh et al. (2004) and Hair et al. (2019), fit indices should not be interpreted as strict pass/fail criteria or considered in isolation when evaluating model fit. In the present studies, the RMSEA values were 0.059 and 0.069, respectively, suggesting a reasonable approximation of the data. Although the CFI values indicate some degree of model misfit, the overall pattern of fit indices provided evidence that the hypothesized factor structure offers a reasonable representation of the data.

## Author’s note

Nathalie Mella and Olivier Desrichard are members of the ProFAN Consortium. Authors Marine Beaudoin, Jérôme Blondé, Fanny Vallet, and Morgane Schlemmer are not members of the ProFAN Consortium.

## Group members of ProFAN Consortium

Batruch Anatolia, Laboratoire de Psychologie Sociale de l’Université de Lausanne—UnilaPS Université Lausanne; Bouet Marinette, Laboratoire d’Informatique de Modélisation et d’Optimisation des Systèmes—LIMOS UMR-6158 Université Clermont Auvergne; Bressan Marco, Laboratoire de Psychologie Cognitive—LPC UMR7290 Aix-Marseille Université; Bressoux Pascal, Laboratoire de Recherche sur les Apprentissages en Contexte—LaRAC EA602 Université Grenoble; Brown Genavee, Laboratoire de Psychologie: Cognition, Comportement, Communication—LP3C EA 1285 Université Rennes; Butera Fabrizio, Laboratoire de Psychologie Sociale de l’Université de Lausanne—UnilaPS Université Lausanne; Cherbonnier Anthony, Laboratoire de Psychologie: Cognition, Comportement, Communication—LP3C EA 1285 Université Rennes; Darnon Céline, Laboratoire de Psychologie Sociale et Cognitive—LAPSCO UMR-6024 Université Clermont Auvergne; Demolliens Marie, Laboratoire de Psychologie Sociale et Cognitive—LAPSCO UMR-6024 Université Clermont Auvergne; De Place Anne-Laure, Laboratoire de Recherche sur les Apprentissages en Contexte—LaRAC EA602 Université Grenoble; Desrichard Olivier, Groupe de recherche en psychologie de la santé, GREPS, Université de Genève; Goron Luc, Laboratoire de Psychologie: Cognition, Comportement, Communication—LP3C EA 1285 Université Rennes; Hemon Brivael, Laboratoire de Psychologie: Cognition, Comportement, Communication—LP3C EA 1285 Université Rennes; Huguet Pascal, Laboratoire de Psychologie Sociale et Cognitive—LAPSCO UMR-6024 Université Clermont Auvergne; Jamet Eric, Laboratoire de Psychologie: Cognition, Comportement, Communication—LP3C EA 1285 Université Rennes; Mazenod Vincent, Laboratoire d’Informatique de Modélisation et d’Optimisation des Systèmes—LIMOS UMR-6158 Université Clermont Auvergne; Mella-Barraco Nathalie, Groupe de recherche en psychologie de la santé, GREPS, Université de Genève; Michinov Estelle, Laboratoire de Psychologie: Cognition, Comportement, Communication—LP3C EA 1285 Université Rennes; Michinov Nicolas, Laboratoire de Psychologie: Cognition, Comportement, Communication—LP3C EA 1285 Université Rennes; Ofosu Nana, Groupe de recherche en psychologie de la santé, GREPS, Université de Genève; Pansu Pascal, Laboratoire de Recherche sur les Apprentissages en Contexte—LaRAC EA602 Université Grenoble; Peter Laurine, Laboratoire de Psychologie: Cognition, Comportement, Communication—LP3C EA 1285 Université Rennes; Poletti Celine, Laboratoire de Psychologie Cognitive—LPC UMR7290 Aix-Marseille Université; Régner Isabelle, Centre de Recherche en Psychologie et Neurosciences—CRPN UMR-7077 Aix-Marseille Université; Riant Mathilde, Laboratoire de Recherche sur les Apprentissages en Contexte—LaRAC EA602 Université Grenoble; Robert Anais, Laboratoire de Psychologie Sociale et Cognitive—LAPSCO UMR-6024 Université Clermont Auvergne; Rudmann Ocyna, Laboratoire de Psychologie Sociale de l’Université de Lausanne—UnilaPS Université Lausanne; Sanrey Camille, Laboratoire de Recherche sur les Apprentissages en Contexte—LaRAC EA602 Université Grenoble; Stanczak Arnaud, Laboratoire de Psychologie Sociale et Cognitive—LAPSCO UMR-6024 Université Clermont Auvergne; Toumani Farouk, Laboratoire d’Informatique de Modélisation et d’Optimisation des Systèmes—LIMOS UMR-6158 Université Clermont Auvergne; Visintin Emilio Paolo, Laboratoire de Psychologie Sociale de l’Université de Lausanne—UnilaPS Université Lausanne; Département de Sciences Humaines, Université de Ferrara; Vives Eva, Laboratoire de Psychologie Cognitive—LPC UMR7290 Aix-Marseille Université.
