# Supplementary material for: The Motivation for Cognitive Activities Questionnaire: scale development and validation in French and English
Source: Front Psychol. 2026 Oct 30;17:1907732. doi: 10.3389/fpsyg.2026.1907732 (PMC13760842; doi:10.3389/fpsyg.2026.1907732)
Supplement: Supplementary file 1 [file Table_1.DOCX]

Supplementary Material

1. **MCAQ (English version)**

This questionnaire looks at the way we perceive the use of our brain to perform everyday activities (personal and professional) **that require concentration and thought.** We start with a general question about activities that use your brain and that require concentration and thought.

Q1.	**How do you rate your ability to use your brain to engage in activities that require concentration or thought?**

*Tick the number, between 1 and 7, that best reflects your opinion. Please tick only one box:*

| *1 = Very poor* | |  | | | | | *7 = Very good* | |
| --- | --- | --- | --- | --- | --- | --- | --- | --- |
|  | 1 | 2 | 3 | 4 | 5 | 6 | 7 |  |
|  | ☐ | ☐ | ☐ | ☐ | ☐ | ☐ | ☐ |  |

We are now going to present several activities you to use your brain and that require concentration and thought (for example, reading). Please think about each activity in terms of how you do it **most of the time, in most cases.** The examples given in *italics* are there just to give you ideas. As everyone is different, we would like you to think about how you do the activity **most of the time in your day-to-day life.**

There are no right or wrong answers. Simply indicate what you think.

Q2.	**For each activity presented below, rate your abilities in these kinds of situations…**

*Tick the number, between 1 and 7, that best reflects your opinion. Please tick only one box on each line:*

| Rate your abilities in these kinds of situations: | *1 = Very poor* |  |  |  |  |  | *7 = Very good* |
| --- | --- | --- | --- | --- | --- | --- | --- |
|  | 1 | 2 | 3 | 4 | 5 | 6 | 7 |
| 2.1. Concentrate and think in order to:  **Analyze and solve problems**  e.g., *compare prices, adapt to unforeseen circumstances, resolve a conflict, etc.* | ☐ | ☐ | ☐ | ☐ | ☐ | ☐ | ☐ |
| 2.2. Concentrate and think in order to:  D**o** **calculations in your head**  e.g., *calculate* *a journey time, the amount of change needed to buy something, the right dose of medicine, an age difference, the total of a bill, etc.* | ☐ | ☐ | ☐ | ☐ | ☐ | ☐ | ☐ |
|  |  |  |  |  |  |  |  |

| Rate your abilities in these kinds of situations: | *1 = Very poor* |  |  |  |  |  | *7 = Very good* |
| --- | --- | --- | --- | --- | --- | --- | --- |
|  | 1 | 2 | 3 | 4 | 5 | 6 | 7 |
| 2.3. Concentrate and think in order to:  **Use your sense of direction**  e.g., *work out where you are in an unknown town, get somewhere, read a map, give directions to someone, etc.* | ☐ | ☐ | ☐ | ☐ | ☐ | ☐ | ☐ |
| 2.4. Concentrate and think in order to:  M**emorize**  e.g., *a shopping list, a medical prescription, people’s names, a song, the title of a book, etc.* | ☐ | ☐ | ☐ | ☐ | ☐ | ☐ | ☐ |
| 2.5. Concentrate and think in order to:  **Remember something**  e.g., *a past event, a name, a story, a nice memory, a piece of information, an appointment, etc.* | ☐ | ☐ | ☐ | ☐ | ☐ | ☐ | ☐ |
| 2.6. Concentrate and think in order to:  P**lan or organize**  e.g., *a trip, a project, a doctor’s appointment, a birthday party, etc.* | ☐ | ☐ | ☐ | ☐ | ☐ | ☐ | ☐ |
|  |  |  |  |  |  |  |  |

| Rate your abilities in these kinds of situations: | *1 = Very poor* |  |  |  |  |  | *7 = Very good* |
| --- | --- | --- | --- | --- | --- | --- | --- |
|  | 1 | 2 | 3 | 4 | 5 | 6 | 7 |
| 2.7. Concentrate and think in order to:  **Succeed on mental ability tests**  e.g., *do mental calculations, memorize a list of words, solve logic problems, do spatial orientation tests, etc.* | ☐ | ☐ | ☐ | ☐ | ☐ | ☐ | ☐ |
| 2.8. Concentrate and think in order to:  R**ead a short texts**  e.g., *a notice, an information leaflet, a recipe, mail, etc.* | ☐ | ☐ | ☐ | ☐ | ☐ | ☐ | ☐ |

We would now like to ask you some more questions about your perceptions of situations requiring concentration or thought. Please answer a general question about your perception of activities that use your brain and require concentration or thought.

Q3.	**Most of the time, for what reason(s) do you engage in activities that require concentration or thought?**

*Tick the number, between 1 and 7, that best reflects your opinion. Please tick only one box on each line:*

|  | *1 =*  *Not at all* |  |  |  |  |  | *7 = Totally* |
| --- | --- | --- | --- | --- | --- | --- | --- |
|  | 1 | 2 | 3 | 4 | 5 | 6 | 7 |
| 3.1. I like it, I enjoy it (satisfaction) | ☐ | ☐ | ☐ | ☐ | ☐ | ☐ | ☐ |
| 3.2. It’s useful to me and/or it’s who I am and/or I do it for my own good | ☐ | ☐ | ☐ | ☐ | ☐ | ☐ | ☐ |
| 3.3. I’m forced to or I think that it’s expected of me | ☐ | ☐ | ☐ | ☐ | ☐ | ☐ | ☐ |

In this section, we are going to look at how often and for what reasons you do these sorts of activities. We will present the same activities as earlier and we would like you to think about each one in terms of how you do it **most of the time, in most cases.** The examples given in *italics* are there just to give you ideas. As everyone is different, we want you to think about how you do the activity **most of the time in your day-to-day life.**

There are no right or wrong answers. Simply indicate what you think.

Concentrate and think in order to: **Analyze and solve problems**

E.g., *compare prices, adapt to unforeseen circumstances, resolve a conflict, etc.*

Q4.	**I do this kind of activity:**

*Tick only one answer:*

☐	Every day

☐	Every week

☐	Every month

☐	More rarely than that

☐	Never	* Go to question Q6***^[1]^**

Q5.	Most of the time, for what reason(s) do you engage in activities that involve **analyzing and solving problems**?

*Tick the number, between 1 and 7, that best reflects your opinion. Please tick only one box on each line:*

|  | *1 =*  *Not at all* | |  |  |  |  | *7 = Totally* |
| --- | --- | --- | --- | --- | --- | --- | --- |
|  | 1 | 2 | 3 | 4 | 5 | 6 | 7 |
| 5.1. I like it, I enjoy it (satisfaction) | ☐ | ☐ | ☐ | ☐ | ☐ | ☐ | ☐ |
| 5.2. It’s useful to me and/or it’s who I am and/or I do it for my own good | ☐ | ☐ | ☐ | ☐ | ☐ | ☐ | ☐ |
| 5.3. I’m forced to or I think that it’s expected of me | ☐ | ☐ | ☐ | ☐ | ☐ | ☐ | ☐ |

Concentrate and think in order to: **Do calculations in your head**

e.g., *calculate* *a journey time, the amount of change needed to buy something, the right dose of medicine, an age difference, the total of a bill, etc.*

Q6.	**I do this type of activity:**

*Tick only one answer:*

☐	Every day

☐	Every week

☐	Every month

☐	More rarely than that

☐	Never	* Go to question Q8*

Q7.	Most of the time, for what reason(s) do you **do calculations in your head**?

*Tick the number, between 1 and 7, that best reflects your opinion. Please tick only one box on each line:*

|  | *1 =*  *Not at all* |  |  |  |  |  | *7 = Totally* |
| --- | --- | --- | --- | --- | --- | --- | --- |
|  | 1 | 2 | 3 | 4 | 5 | 6 | 7 |
| 7.1. I like it, I enjoy it (satisfaction) | ☐ | ☐ | ☐ | ☐ | ☐ | ☐ | ☐ |
| 7.2. It’s useful to me and/or it’s who I am and/or I do it for my own good | ☐ | ☐ | ☐ | ☐ | ☐ | ☐ | ☐ |
| 7.3. I’m forced to or I think that it’s expected of me | ☐ | ☐ | ☐ | ☐ | ☐ | ☐ | ☐ |

Concentrate and think in order to: **Use your sense of direction**

e.g., *work out where you are in an unknown town, get somewhere, read a map, give directions to someone, etc.*

Q8.	**I do this type of activity:**

*Tick only one answer:*

☐	Every day

☐	Every week

☐	Every month

☐	More rarely than that

☐	Never	* Go to question Q10*

Q9.	Most of the time, for what reason(s) do you use your **sense of direction**?

*Tick the number, between 1 and 7, that best reflects your opinion. Please tick only one box on each line:*

|  | *1 =*  *Not at all* |  |  |  |  |  | *7 = Totally* |
| --- | --- | --- | --- | --- | --- | --- | --- |
|  | 1 | 2 | 3 | 4 | 5 | 6 | 7 |
| 9.1. I like it, I enjoy it (satisfaction) | ☐ | ☐ | ☐ | ☐ | ☐ | ☐ | ☐ |
| 9.2. It’s useful to me and/or it’s who I am and/or I do it for my own good | ☐ | ☐ | ☐ | ☐ | ☐ | ☐ | ☐ |
| 9.3. I’m forced to or I think that it’s expected of me | ☐ | ☐ | ☐ | ☐ | ☐ | ☐ | ☐ |

Concentrate and think in order to: **Memorize**

e.g., *a shopping list, a medical prescription, people’s names, a song, the title of a book, etc.*

Q10.	**I do this type of activity:**

*Tick only one answer:*

☐	Every day

☐	Every week

☐	Every month

☐	More rarely than that

☐	Never	* Go to question Q12*

Q11.	Most of the time, for what reason(s) do you **memorize things**?

*Tick the number, between 1 and 7, that best reflects your opinion. Please tick only one box on each line:*

|  | *1 =*  *Not at all* |  |  |  |  |  | *7 = Totally* |
| --- | --- | --- | --- | --- | --- | --- | --- |
|  | 1 | 2 | 3 | 4 | 5 | 6 | 7 |
| 11.1. I like it, I enjoy it (satisfaction) | ☐ | ☐ | ☐ | ☐ | ☐ | ☐ | ☐ |
| 11.2. It’s useful to me and/or it’s who I am and/or I do it for my own good | ☐ | ☐ | ☐ | ☐ | ☐ | ☐ | ☐ |
| 11.3. I’m forced to or I think that it’s expected of me | ☐ | ☐ | ☐ | ☐ | ☐ | ☐ | ☐ |

Concentrate and think in order to: **Remember something**

e.g., *a past event, a name, a story, a nice memory, a piece of information, an appointment, etc.*

Q12.	**I do this type of activity:**

*Tick only one answer:*

☐	Every day

☐	Every week

☐	Every month

☐	More rarely than that

☐	Never	* Go to question Q14*

Q13.	Most of the time, for what reason(s) do you **remember something**?

*Tick the number, between 1 and 7, that best reflects your opinion. Please tick only one box on each line:*

|  | *1 =*  *Not at all* |  |  |  |  |  | *7 = Totally* |
| --- | --- | --- | --- | --- | --- | --- | --- |
|  | 1 | 2 | 3 | 4 | 5 | 6 | 7 |
| 13.1. I like it, I enjoy it (satisfaction) | ☐ | ☐ | ☐ | ☐ | ☐ | ☐ | ☐ |
| 13.2. It’s useful to me and/or it’s who I am and/or I do it for my own good | ☐ | ☐ | ☐ | ☐ | ☐ | ☐ | ☐ |
| 13.3. I’m forced to or I think that it’s expected of me | ☐ | ☐ | ☐ | ☐ | ☐ | ☐ | ☐ |

Concentrate and think in order to: **Plan or organize**

E.g., *a trip, a project, a doctor’s appointment, a birthday party, etc.*

Q 14.	**I do this type of activity:**

*Tick only one answer:*

☐	Every day

☐	Every week

☐	Every month

☐	More rarely than that

☐	Never	* Go to question Q16*

Q15.	Most of the time, for what reason(s) do you **plan or organize**?

*Tick the number, between 1 and 7, that best reflects your opinion. Please tick only one box on each line:*

|  | *1 =*  *Not at all* |  |  |  |  |  | *7 = Totally* |
| --- | --- | --- | --- | --- | --- | --- | --- |
|  | 1 | 2 | 3 | 4 | 5 | 6 | 7 |
| 15.1. I like it, I enjoy it (satisfaction) | ☐ | ☐ | ☐ | ☐ | ☐ | ☐ | ☐ |
| 15.2. It’s useful to me and/or it’s who I am and/or I do it for my own good | ☐ | ☐ | ☐ | ☐ | ☐ | ☐ | ☐ |
| 15.3. I’m forced to or I think that it’s expected of me | ☐ | ☐ | ☐ | ☐ | ☐ | ☐ | ☐ |

Concentrate and think in order to: **Succeed on mental ability tests**

e.g., *do mental calculations, memorize a list of words, solve logic problems, do spatial orientation tests, etc.*

Q16.	**I do this type of activity:**

*Tick only one answer:*

☐	Every day

☐	Every week

☐	Every month

☐	More rarely than that

☐	Never	* Go to question Q18*

Q17.	Most of the time, for what reason(s) do you **do mental ability tests**?

*Tick the number, between 1 and 7, that best reflects your opinion. Please tick only one box on each line:*

|  | *1 =*  *Not at all* |  |  |  |  |  | *7 = Totally* |
| --- | --- | --- | --- | --- | --- | --- | --- |
|  | 1 | 2 | 3 | 4 | 5 | 6 | 7 |
| 17.1. I like it, I enjoy it (satisfaction) | ☐ | ☐ | ☐ | ☐ | ☐ | ☐ | ☐ |
| 17.2. It’s useful to me and/or it’s who I am and/or I do it for my own good | ☐ | ☐ | ☐ | ☐ | ☐ | ☐ | ☐ |
| 17.3. I’m forced to or I think that it’s expected of me | ☐ | ☐ | ☐ | ☐ | ☐ | ☐ | ☐ |

Concentrate and think in order to: **Read a short texts**

E.g., *a notice, an information leaflet, a recipe, mail, etc.*

Q18.	**I do this type of activity:**

*Tick only one answer:*

☐	Every day

☐	Every week

☐	Every month

☐	More rarely than that

☐	Never

Q19.	Most of the time, for what reason(s) do you **read a short text**?

*Tick the number, between 1 and 7, that best reflects your opinion. Please tick only one box on each line:*

|  | *1 =*  *Not at all* |  |  |  |  |  | *7 = Totally* |
| --- | --- | --- | --- | --- | --- | --- | --- |
|  | 1 | 2 | 3 | 4 | 5 | 6 | 7 |
| 19.1. I like it, I enjoy it (satisfaction) | ☐ | ☐ | ☐ | ☐ | ☐ | ☐ | ☐ |
| 19.2. It’s useful to me and/or it’s who I am and/or I do it for my own good | ☐ | ☐ | ☐ | ☐ | ☐ | ☐ | ☐ |
| 19.3. I’m forced to or I think that it’s expected of me | ☐ | ☐ | ☐ | ☐ | ☐ | ☐ | ☐ |

2. **MCAQ (French version)**

Le présent questionnaire s’intéresse à la façon dont nous percevons l'utilisation de notre cerveau dans la vie quotidienne pour effectuer des activités (personnelles et professionnelles) **qui mobilisent la concentration et la réflexion.** Merci d'abord de répondre à une question générale sur les activités qui font travailler votre cerveau et qui mobilisent la concentration et la réflexion.

Q1.	**Estimez vos capacités à utiliser votre cerveau pour vous engager dans des activités qui mobilisent la concentration ou la réflexion…**

*Indiquez le nombre entre 1 et 7 qui reflète le mieux votre avis. Veuillez cocher une seule case :*

| *1 = Très mauvaises* | |  | | | | | *7 = Très bonnes* | |
| --- | --- | --- | --- | --- | --- | --- | --- | --- |
|  | 1 | 2 | 3 | 4 | 5 | 6 | 7 |  |
|  | ☐ | ☐ | ☐ | ☐ | ☐ | ☐ | ☐ |  |

Maintenant nous allons vous présenter plusieurs de ces activités qui font travailler votre cerveau (par exemple : lire). Vous devez penser à chaque activité telle que vous la pratiquez **la plupart du temps, dans la majorité des cas.** Les exemples donnés en *italiques* ne servent qu'à donner des idées. Comme chaque personne est différente, c’est à vous de penser à votre manière de pratiquer cette activité **la plupart du temps dans votre vie quotidienne.**

Il n’y a pas de bonnes ou de mauvaises réponses. Indiquez simplement ce que vous pensez.

Q2.	**Pour chaque activité présentée ci-dessous, estimez vos capacités dans ce genre de situations…**

*Indiquez le nombre entre 1 et 7 qui reflète le mieux votre avis. Veuillez cocher une case par ligne :*

| Estimez vos capacités dans ce genre de situations : | *1 = Très mauvaises capacités* | |  |  |  | *7 = Très bonnes capacités* | |
| --- | --- | --- | --- | --- | --- | --- | --- |
|  | 1 | 2 | 3 | 4 | 5 | 6 | 7 |
| 2.1. Se concentrer et réfléchir pour :  **Analyser et trouver la solution à un problème**  Par ex. *comparer des prix, s'adapter à une situation imprévue, résoudre un conflit…* | ☐ | ☐ | ☐ | ☐ | ☐ | ☐ | ☐ |
| 2.2. Se concentrer et réfléchir pour :  **Calculer de tête**  Par ex. *un temps de trajet, la monnaie pour les courses, le dosage des médicaments, une différence d’âge, le total d’une facture…* | ☐ | ☐ | ☐ | ☐ | ☐ | ☐ | ☐ |

| Estimez vos capacités dans ce genre de situations : | *1 = Très mauvaises capacités* | | |  | |  | |  | | *7 = Très bonnes capacités* | | | |  |
| --- | --- | --- | --- | --- | --- | --- | --- | --- | --- | --- | --- | --- | --- | --- |
|  | 1 | 2 | | 3 | | 4 | | 5 | | 6 | | 7 | |  |
| 2.3. Se concentrer et réfléchir pour :  **S'orienter dans l'espace**  Par ex*. pour me repérer dans une ville que je ne connais pas, pour aller quelque part, pour lire un plan, pour expliquer un chemin…* | ☐ | ☐ | | ☐ | | ☐ | | ☐ | | ☐ | | ☐ | |  |
| 2.4. Se concentrer et réfléchir pour :  **Mémoriser**  Par ex. *une liste de course, la prescription d'un médecin, les noms des gens, une chanson, un titre de livre…* | ☐ | | ☐ | | ☐ | | ☐ | | ☐ | | ☐ | | ☐ | |
| 2.5. Se concentrer et réfléchir pour :  **Se rappeler quelque chose**  Par ex. *d’un événement passé, d'un nom, d’une histoire, d'un bon souvenir, d’une information précise, d'un rendez-vous…* | ☐ | | ☐ | | ☐ | | ☐ | | ☐ | | ☐ | | ☐ | |
| 2.6. Se concentrer et réfléchir pour :  **Planifier, organiser**  Par ex. *un déplacement, un projet, un rendez-vous médical, un anniversaire…* | ☐ | | ☐ | | ☐ | | ☐ | | ☐ | | ☐ | | ☐ | |

| Estimez vos capacités dans ce genre de situations : | *1 = Très mauvaises capacités* | |  | |  | |  | | *7 = Très bonnes capacités* | | |
| --- | --- | --- | --- | --- | --- | --- | --- | --- | --- | --- | --- |
|  | 1 | 2 | | 3 | | 4 | | 5 | | 6 | 7 |
| 2.7. Se concentrer et réfléchir pour :  **Réussir des tests de capacités intellectuelles**  Par ex. *faire des calculs mentaux, mémoriser une liste de mots, faire des exercices de logique, faire des tests d'orientation dans l'espace...* | ☐ | ☐ | | ☐ | | ☐ | | ☐ | | ☐ | ☐ |
| 2.8. Se concentrer et réfléchir pour :  **Lire un texte court**  Par ex. *une notice, une information, une recette, un courrier...* | ☐ | ☐ | | ☐ | | ☐ | | ☐ | | ☐ | ☐ |

A présent nous allons vous poser de nouvelles questions au sujet de votre perception des situations qui mobilisent la concentration ou la réflexion.

Merci de répondre à une question générale sur votre perception des activités qui font travailler votre cerveau.

Q3.	**La plupart du temps, pour quelle(s) raison(s) vous engagez-vous dans des activités qui mobilisent la concentration ou la réflexion ?**

*Indiquez le nombre entre 1 et 7 qui reflète le mieux votre avis. Veuillez cocher une case par ligne :*

|  | *1 =*  *Pas du tout* |  |  |  |  |  | *7 = Tout à fait* |
| --- | --- | --- | --- | --- | --- | --- | --- |
|  | 1 | 2 | 3 | 4 | 5 | 6 | 7 |
| 3.1. Ça me plaît, j’y prends du plaisir (satisfaction) | ☐ | ☐ | ☐ | ☐ | ☐ | ☐ | ☐ |
| 3.2. Ça m’est utile et/ou ça me correspond et/ou je le fais pour mon bien | ☐ | ☐ | ☐ | ☐ | ☐ | ☐ | ☐ |
| 3.3. J’y suis contraint(e) ou je pense que c’est ce que l’on attend de moi | ☐ | ☐ | ☐ | ☐ | ☐ | ☐ | ☐ |

Dans cette partie l'on va s'intéresser à la fréquence et aux raisons pour lesquelles on fait des activités. Les mêmes activités que précédemment vont être présentées, et vous devez penser à chaque activité, telle que vous la pratiquez **la plupart du temps, dans la majorité des cas.** Les exemples donnés en *italiques* ne servent qu’à donner des idées. Comme chaque personne est différente, c’est à vous de penser à votre manière de pratiquer cette activité **la plupart du temps dans votre vie quotidienne.**

Il n’y a pas de bonnes ou de mauvaises réponses. Indiquez simplement ce que vous pensez.

Se concentrer et réfléchir pour : **Analyser et trouver la solution à un problème**

Par ex. *comparer des prix, s’adapter à une situation imprévue, résoudre un conflit…*

Q4.	**Je fais ce genre d’activité :**

*Veuillez cocher une seule proposition :*

☐	Tous les jours

☐	Toutes les semaines

☐	Tous les mois

☐	Plus rarement que ça

☐	Jamais	* Passez à la question Q6*

Q5.	La plupart du temps pour quelle(s) raison(s) vous engagez-vous dans des activités d’**analyse et de résolution de problèmes** ?

*Indiquez le nombre entre 1 et 7 qui reflète le mieux votre avis. Veuillez cocher une case par ligne :*

|  | *1 =*  *Pas du tout* |  |  |  |  |  | *7 = Tout à fait* |
| --- | --- | --- | --- | --- | --- | --- | --- |
|  | 1 | 2 | 3 | 4 | 5 | 6 | 7 |
| 5.1. Ça me plaît, j’y prends du plaisir (satisfaction) | ☐ | ☐ | ☐ | ☐ | ☐ | ☐ | ☐ |
| 5.2. Ça m’est utile et/ou ça me correspond et/ou je le fais pour mon bien | ☐ | ☐ | ☐ | ☐ | ☐ | ☐ | ☐ |
| 5.3. J’y suis contraint(e) ou je pense que c’est ce que l’on attend de moi | ☐ | ☐ | ☐ | ☐ | ☐ | ☐ | ☐ |

Se concentrer et réfléchir pour : **Calculer de tête**

Par ex. *un temps de trajet, la monnaie pour les courses, le dosage des médicaments, une différence d’âge, le total d’une facture*…

Q6.	**Je fais ce genre d’activité :**

*Veuillez cocher une seule proposition :*

☐	Tous les jours

☐	Toutes les semaines

☐	Tous les mois

☐	Plus rarement que ça

☐	Jamais	* Passez à la question Q8*

Q7.	La plupart du temps pour quelle(s) raison(s) **calculez-vous quelque chose de tête** ?

*Indiquez le nombre entre 1 et 7 qui reflète le mieux votre avis. Veuillez cocher une case par ligne :*

|  | *1 =*  *Pas du tout* |  |  |  |  |  | *7 = Tout à fait* |
| --- | --- | --- | --- | --- | --- | --- | --- |
|  | 1 | 2 | 3 | 4 | 5 | 6 | 7 |
| 7.1. Ça me plaît, j’y prends du plaisir (satisfaction) | ☐ | ☐ | ☐ | ☐ | ☐ | ☐ | ☐ |
| 7.2. Ça m’est utile et/ou ça me correspond et/ou je le fais pour mon bien | ☐ | ☐ | ☐ | ☐ | ☐ | ☐ | ☐ |
| 7.3. J’y suis contraint(e) ou je pense que c’est ce que l’on attend de moi | ☐ | ☐ | ☐ | ☐ | ☐ | ☐ | ☐ |

Se concentrer et réfléchir pour : **S'orienter dans l'espace**

Par ex. *pour me repérer dans une ville que je ne connais pas, pour aller quelque part, pour lire un plan, pour expliquer un chemin…*

Q8.	**Je fais ce genre d’activité :**

*Veuillez cocher une seule proposition :*

☐	Tous les jours

☐	Toutes les semaines

☐	Tous les mois

☐	Plus rarement que ça

☐	Jamais	* Passez à la question Q10*

Q9.	La plupart du temps pour quelle(s) raison(s) utilisez-vous votre **sens de l’orientation** ?

*Indiquez le nombre entre 1 et 7 qui reflète le mieux votre avis. Veuillez cocher une case par ligne :*

|  | *1 =*  *Pas du tout* |  |  |  |  |  | *7 = Tout à fait* |
| --- | --- | --- | --- | --- | --- | --- | --- |
|  | 1 | 2 | 3 | 4 | 5 | 6 | 7 |
| 9.1. Ça me plaît, j’y prends du plaisir (satisfaction) | ☐ | ☐ | ☐ | ☐ | ☐ | ☐ | ☐ |
| 9.2. Ça m’est utile et/ou ça me correspond et/ou je le fais pour mon bien | ☐ | ☐ | ☐ | ☐ | ☐ | ☐ | ☐ |
| 9.3. J’y suis contraint(e) ou je pense que c’est ce que l’on attend de moi | ☐ | ☐ | ☐ | ☐ | ☐ | ☐ | ☐ |

Se concentrer et réfléchir pour : **Mémoriser**

Par ex. *une liste de course, la prescription d’un médecin, les noms des gens, une chanson, un titre de livre…*

Q10.	**Je fais ce genre d’activité :**

*Veuillez cocher une seule proposition :*

☐	Tous les jours

☐	Toutes les semaines

☐	Tous les mois

☐	Plus rarement que ça

☐	Jamais	* Passez à la question Q12*

Q11.	La plupart du temps pour quelle(s) raison(s) **mémorisez-vous quelque chose** ?

*Indiquez le nombre entre 1 et 7 qui reflète le mieux votre avis. Veuillez cocher une case par ligne :*

|  | *1 =*  *Pas du tout* |  |  |  |  |  | *7 = Tout à fait* |
| --- | --- | --- | --- | --- | --- | --- | --- |
|  | 1 | 2 | 3 | 4 | 5 | 6 | 7 |
| 11.1. Ça me plaît, j’y prends du plaisir (satisfaction) | ☐ | ☐ | ☐ | ☐ | ☐ | ☐ | ☐ |
| 11.2. Ça m’est utile et/ou ça me correspond et/ou je le fais pour mon bien | ☐ | ☐ | ☐ | ☐ | ☐ | ☐ | ☐ |
| 11.3. J’y suis contraint(e) ou je pense que c’est ce que l’on attend de moi | ☐ | ☐ | ☐ | ☐ | ☐ | ☐ | ☐ |

Se concentrer et réfléchir pour : **Se rappeler quelque chose**

Par ex. *d’un événement passé, d’un nom, d’une histoire, d’un bon souvenir, d’une information précise, d’un rendez-vous…*

Q12.	**Je fais ce genre d’activité :**

*Veuillez cocher une seule proposition :*

☐	Tous les jours

☐	Toutes les semaines

☐	Tous les mois

☐	Plus rarement que ça

☐	Jamais	* Passez à la question Q14*

Q13.	La plupart du temps pour quelle(s) raison(s) cherchez-vous à vous **souvenir de quelque chose** ?

*Indiquez le nombre entre 1 et 7 qui reflète le mieux votre avis. Veuillez cocher une case par ligne :*

|  | *1 =*  *Pas du tout* |  |  |  |  |  | *7 = Tout à fait* |
| --- | --- | --- | --- | --- | --- | --- | --- |
|  | 1 | 2 | 3 | 4 | 5 | 6 | 7 |
| 13.1. Ça me plaît, j’y prends du plaisir (satisfaction) | ☐ | ☐ | ☐ | ☐ | ☐ | ☐ | ☐ |
| 13.2. Ça m’est utile et/ou ça me correspond et/ou je le fais pour mon bien | ☐ | ☐ | ☐ | ☐ | ☐ | ☐ | ☐ |
| 13.3. J’y suis contraint(e) ou je pense que c’est ce que l’on attend de moi | ☐ | ☐ | ☐ | ☐ | ☐ | ☐ | ☐ |

Se concentrer et réfléchir pour : **Planifier, organiser**

Par ex. *un déplacement, un projet, un rendez-vous médical, un anniversaire…*

Q 14.	**Je fais ce genre d’activité :**

*Veuillez cocher une seule proposition :*

☐	Tous les jours

☐	Toutes les semaines

☐	Tous les mois

☐	Plus rarement que ça

☐	Jamais	* Passez à la question Q16*

Q15.	La plupart du temps pour quelle(s) raison(s) vous mettez-vous à **planifier, organiser les choses** ?

*Indiquez le nombre entre 1 et 7 qui reflète le mieux votre avis. Veuillez cocher une case par ligne :*

|  | *1 =*  *Pas du tout* |  |  |  |  |  | *7 = Tout à fait* |
| --- | --- | --- | --- | --- | --- | --- | --- |
|  | 1 | 2 | 3 | 4 | 5 | 6 | 7 |
| 15.1. Ça me plaît, j’y prends du plaisir (satisfaction) | ☐ | ☐ | ☐ | ☐ | ☐ | ☐ | ☐ |
| 15.2. Ça m’est utile et/ou ça me correspond et/ou je le fais pour mon bien | ☐ | ☐ | ☐ | ☐ | ☐ | ☐ | ☐ |
| 15.3. J’y suis contraint(e) ou je pense que c’est ce que l’on attend de moi | ☐ | ☐ | ☐ | ☐ | ☐ | ☐ | ☐ |

Se concentrer et réfléchir pour : **Réussir des tests de capacités intellectuelles**

Par ex. *faire des calculs mentaux, mémoriser une liste de mots, faire des exercices de logique, faire des tests d’orientation dans l’espace…*

Q16.	**Je fais ce genre d’activité :**

*Veuillez cocher une seule proposition :*

☐	Tous les jours

☐	Toutes les semaines

☐	Tous les mois

☐	Plus rarement que ça

☐	Jamais	* Passez à la question Q18*

Q17.	La plupart du temps pour quelle(s) raison(s) faites-vous des **tests pour évaluer vos capacités intellectuelles** ?

*Indiquez le nombre entre 1 et 7 qui reflète le mieux votre avis. Veuillez cocher une case par ligne :*

|  | *1 =*  *Pas du tout* |  |  |  |  |  | *7 = Tout à fait* |
| --- | --- | --- | --- | --- | --- | --- | --- |
|  | 1 | 2 | 3 | 4 | 5 | 6 | 7 |
| 17.1. Ça me plaît, j’y prends du plaisir (satisfaction) | ☐ | ☐ | ☐ | ☐ | ☐ | ☐ | ☐ |
| 17.2. Ça m’est utile et/ou ça me correspond et/ou je le fais pour mon bien | ☐ | ☐ | ☐ | ☐ | ☐ | ☐ | ☐ |
| 17.3. J’y suis contraint(e) ou je pense que c’est ce que l’on attend de moi | ☐ | ☐ | ☐ | ☐ | ☐ | ☐ | ☐ |

Se concentrer et réfléchir pour : **Lire un texte court**

Par ex. *une notice, une information, une recette, un courrier...*

Q18.	**Je fais ce genre d’activité :**

*Veuillez cocher une seule proposition :*

☐	Tous les jours

☐	Toutes les semaines

☐	Tous les mois

☐	Plus rarement que ça

☐	Jamais

Q19.	La plupart du temps pour quelle(s) raison(s) **lisez-vous un texte court** ?

*Indiquez le nombre entre 1 et 7 qui reflète le mieux votre avis. Veuillez cocher une case par ligne :*

|  | *1 =*  *Pas du tout* |  |  |  |  |  | *7 = Tout à fait* |
| --- | --- | --- | --- | --- | --- | --- | --- |
|  | 1 | 2 | 3 | 4 | 5 | 6 | 7 |
| 19.1. Ça me plaît, j’y prends du plaisir (satisfaction) | ☐ | ☐ | ☐ | ☐ | ☐ | ☐ | ☐ |
| 19.2. Ça m’est utile et/ou ça me correspond et/ou je le fais pour mon bien | ☐ | ☐ | ☐ | ☐ | ☐ | ☐ | ☐ |
| 19.3. J’y suis contraint(e) ou je pense que c’est ce que l’on attend de moi | ☐ | ☐ | ☐ | ☐ | ☐ | ☐ | ☐ |

3. **Multigroup CFAs (Study 3)**

We assessed the measurement invariance of the MCAQ across languages using multigroup confirmatory factor analysis. This involved testing a series of increasingly constrained structural equation models. Model 1 tested configural invariance: no parameters were constrained across languages. This model fitted the data well, χ²(820; n = 435) = 1534.172, p < .05, CFI = .902, RMSEA = .045. Model 2 tested metric invariance: factor loadings were constrained to be equal across languages. This model also fitted well to the data, χ²(848; n = 435) = 1569.220, p < .05, CFI = .901, RMSEA = .044), and did not differ significantly from Model 1 (∆CFI < .01; ∆RMSEA < .015), indicating that factor loadings did not differ between languages. Model 3 tested scalar invariance: both factor loadings and intercepts were constrained to be equal. This model fitted the data adequately (χ²(880; n = 435) = 1649.029, p < .05, CFI = .895, RMSEA = .045), and did not differ significantly from Model 1 (∆CFI < .01; ∆RMSEA < .015), demonstrating scalar invariance. Therefore, our results showed that the factor structure of the MCAQ is invariant across languages, with both the meaning of the construct (factor loadings) and the levels of the underlying items (intercepts) being equivalent across groups.

1. **Test-Retest Statistics (Study 3)**

**Table S1.** Test-Retest Statistics (English/French speaking participants), Study 3

|  |  | **Test**  **Mean T1** | **Retest**  **Mean T2** | **Correlation (T1-T2)*** | **Significance of**  ***t*-value** |
| --- | --- | --- | --- | --- | --- |
| General items | Perceived capacity | 5.84 / 5.32 | 5.88 / 5.27 | .56 / .55 | .71 / .41 |
|  | Intrinsic | 5.28 / 5.44 | 5.30 / 5.31 | .67 / .51 | .88 / .16 |
|  | Identified | 5.78 / 5.65 | 5.91 / 5.6 | .38 / .39 | .27 / .61 |
|  | External | 3.79 / 3.48 | 4.18 / 3.64 | .37 / .58 | .04** / .18 |
| Perceived capacity - Activity-specific dimensions | Solve Problems | 5.98 / 5.68 | 5.87 / 5.61 | .52 / .48 | .21 / .40 |
|  | Calculate | 5.32 / 5.05 | 5.27 / 5.13 | .67 / .74 | .56 / .30 |
|  | Sense of Direction | 5.18 / 4.90 | 5.23 / 5.02 | .64 / .78 | .65 / .11 |
|  | Memorize | 5.17 / 4.88 | 5.06 / 4.89 | .66 / .62 | .23 / .89 |
|  | Remember | 5.46 / 5.13 | 5.36 / 5.07 | .55 / .62 | .31 / .52 |
|  | Organize | 5.95 / 5.77 | 5.91 / 5.80 | .50 / .54 | .62 / .70 |
|  | Succeed in tests | 5.20 / 5.05 | 5.23 / 5.14 | .53 / .58 | .76 / .23 |
|  | Read | 6.2 / 6.06 | 6.23 / 6.11 | .34 / .50 | .69 / .55 |
|  | Subscale | 5.56 / 5.31 | 5.52 / 5.35 | .65 / .70 | .52 / .45 |
| Intrinsic motivation -  Activity-specific dimensions | Solve Problems | 5.13 / 4.95 | 4.89 / 4.99 | .55 / .54 | .06 / .70 |
|  | Calculate | 4.49 / 4.16 | 4.34 / 4.36 | .57 / .75 | .29 / .04 |
|  | Sense of Direction | 4.34 / 4.26 | 4.23 / 4.31 | .64 / .73 | .41 / .61 |
|  | Memorize | 4.33 / 4.67 | 4.43 / 4.62 | .56 / .57 | .48 / .64 |
|  | Remember | 5.11 / 4.82 | 5.09 / 4.91 | .46 / .43 | .92 / .47 |
|  | Organize | 5.06 / 4.82 | 4.87 / 5.21 | .57 / .60 | .21 / .00** |
|  | Succeed in Tests | 4.71 / 4.9 | 4.61 / 4.94 | .63 / .53 | .49 / .77 |
|  | Read | 5.65 / 5.47 | 5.33 / 5.37 | .44 / .47 | .03** / .40 |
|  | Subscale | 4.84 / 4.75 | 4.71 / 4.82 | .73 / .69 | .10 / .20 |
| Identified motivation -  Activity-specific dimensions | Solve Problems | 5.95 / 5.39 | 5.93 / 5.55 | .54 / .41 | .81 / .12 |
|  | Calculate | 5.84 / 5.37 | 5.89 / 5.49 | .36 / .51 | .67 / .23 |
|  | Sense of Direction | 5.70 / 5.29 | 5.86 / 5.35 | .36 / .49 | .26 / .51 |
|  | Memorize | 5.80 / 5.52 | 5.71 / 5.43 | .19 / .49 | .56 / .36 |
|  | Remember | 5.88 / 5.35 | 6.04 / 5.6 | .30 / .36 | .19 / .02** |
|  | Organize | 5.93 / 5.53 | 5.87 / 5.73 | .40 / .46 | .59 / .05** |
|  | Succeed in Tests | 5.26 / 4.64 | 5 / 4.75 | .36 / .49 | .11 / .43 |
|  | Read | 6.13 / 5.87 | 6.06 / 5.81 | .40 / .42 | .53 / .57 |
|  | Subscale | 5.80 / 5.37 | 5.79 / 5.46 | .49 / .57 | .89 / .12 |
| External motivation -  Activity-specific dimensions | Solve Problems | 3.98 / 3.94 | 4.34 / 3.97 | .54 / .53 | .03 / .83 |
|  | Calculate | 3.91 / 4.16 | 4.17 / 4.03 | .38 / .47 | .19 / .39 |
|  | Sense of Direction | 3.76 / 4.02 | 3.98 / 3.85 | .36 / .50 | .28 / .23 |
|  | Memorize | 3.77 / 3.84 | 3.96 / 3.75 | .40 / .54 | .32 / .50 |
|  | Remember | 3.8 / 3.81 | 4.13 / 3.94 | .27 / .51 | .13 / .33 |
|  | Organize | 4.06 / 4.15 | 4.28 / 4.18 | .49 / .61 | .23 / .86 |
|  | Succeed in Tests | 3.59 / 3.18 | 3.77 / 3.17 | .49 / .36 | .30 / .94 |
|  | Read | 3.91 / 3.61 | 4 / 3.56 | .57 / .57 | .61 / .75 |
|  | Subscale | 3.85 / 3.84 | 4.06 / 3.82 | .57 / .64 | .10 / .83 |

*Initial and second administration, all significant at 0.05 level.

** Significant at 0.05 level.

2. **Test-Retest Statistics (Study 4)**

**Table S2.** Test-Retest Statistics of the MCAQ, Study 4

|  |  | **Test**  **Mean Time 1** | **Retest**  **Mean Time 2** | **Correlation (T1-T2)** | ***t*-value** | **Hedges’*g*** |
| --- | --- | --- | --- | --- | --- | --- |
| General item | Perceived capacity  Cohort 1  Cohort 2 | 4.64  4.60 | 4.55  4.50 | .40***  .44*** | 3.69***  4.50*** | .06  .04 |
|  | Intrinsic  Cohort 1  Cohort 2 | 4.35  4.25 | 4.13  4.11 | .33***  .31*** | 7.26***  4.50*** | .12  .04 |
|  | Identified  Cohort 1  Cohort 2 | 4.54  4.48 | 4.39  4.36 | .31***  .32*** | 5.36***  4.36*** | .09  .04 |
|  | External  Cohort 1  Cohort 2 | 4.22  4.24 | 4.17  4.13 | .20**  .17*** | 1.39  3.59*** | .02  .03 |
| Perceived capacity - Activity-specific dimensions | Cohort 1  Cohort 2 | 4.85  4.84 | 4.71  4.66 | .47***  .48*** | 8.12***  10.29*** | .13  .17 |
| Intrinsic motivation -  Activity-specific dimensions | Cohort 1  Cohort 2 | 4.01  4.05 | 4.03  4.05 | .53***  .51*** | -0.94  -0.46 | -.02  -.01 |
| Identified motivation -  Activity-specific dimensions | Cohort 1  Cohort 2 | 4.46  4.41 | 4.37  4.33 | .41***  .3*** | 4.68***  4.07*** | .08  .07 |
| External motivation -  Activity-specific dimensions | Cohort 1  Cohort 2 | 3.89  3.93 | 3.95  3.93 | .33**  .28*** | -2.68**  -0.02 | -.04  .01 |

*Note*. *** *p* > .001, ** *p* > .01

1. Note that participants must have performed the activity at least occasionally to be asked about their reasons for engaging in it. If the ‘never’ option is selected, they are subsequently directed to the next activity.
